# A fast SCOP fold classification system using content-based E-Predict algorithm

**DOI:** 10.1186/1471-2105-7-362

**Published:** 2006-07-26

**Authors:** Pin-Hao Chi, Chi-Ren Shyu, Dong Xu

**Affiliations:** 1 Medical and Biological Digital Library Research Lab, Department of Computer Science, University of Missouri, Columbia, MO 65211, USA; 2 Digital Biology Laboratory, Department of Computer Science and Life Sciences Center, University of Missouri, Columbia, MO 65211, USA

## Abstract

**Background:**

Domain experts manually construct the Structural Classification of Protein (SCOP) database to categorize and compare protein structures. Even though using the SCOP database is believed to be more reliable than classification results from other methods, it is labor intensive. To mimic human classification processes, we develop an automatic SCOP fold classification system to assign possible known SCOP folds and recognize novel folds for newly-discovered proteins.

**Results:**

With a sufficient amount of ground truth data, our system is able to assign the known folds for newly-discovered proteins in the latest SCOP *v*1.69 release with 92.17% accuracy. Our system also recognizes the novel folds with 89.27% accuracy using 10 fold cross validation. The average response time for proteins with 500 and 1409 amino acids to complete the classification process is 4.1 and 17.4 seconds, respectively. By comparison with several structural alignment algorithms, our approach outperforms previous methods on both the classification accuracy and efficiency.

**Conclusion:**

In this paper, we build an advanced, non-parametric classifier to accelerate the manual classification processes of SCOP. With satisfactory ground truth data from the SCOP database, our approach identifies relevant domain knowledge and yields reasonably accurate classifications. Our system is publicly accessible at .

## Background

Protein structure classification is well-known to be an important research topic in computational and molecular biology. Through the use of structural classification, life science researchers and biologists are able to study evolutionary evidence from similar proteins that have been conserved in multiple species. In addition, similar 3-D conformations of enzyme active sites and binding sites may correlate with biochemical functions [1]. In recent years, structural genomics projects [2-5] have aimed to link protein sequences to possible functions via high-throughput techniques such as X-ray crystallography and nuclear magnetic resonance (NMR) that determine 3-D protein structures. With a large-scale set of newly-discovered structures, a system that classifies similar protein structures with high efficiency and accuracy becomes an indispensable requirement to the study of structure-to-function relationships.

Several classification systems categorize proteins based on structural similarities. The Class, Architecture, Topology, Homologous Superfamily (CATH) database [6] is constructed by applying the Secondary Structure Alignment Program (SSAP) [7], which consists of a double dynamic programming technique to find the optimal structural alignment of two proteins. The Fold Classification based on Structure-Structure Alignment of Proteins (FSSP) database [8] is built based on the Distance Alignment (DALI) [9] algorithm that applies Monte Carlo heuristics to compare structural similarities from 2-D distance matrices mapped from 3-D protein structures. Generally, these systems rely on the structural alignment algorithms to measure the similarity of two proteins, which is known to be of complexity NP-Hard [10]. To reduce the computational effort of scanning large-scale protein databases, those structural alignment algorithms need to apply heuristics with trade-offs which may return divergent results from the same query protein. At present, the Structural Classification of Protein (SCOP) database [11], which is manually constructed by human experts, is believed to contain the most accurate structural classifications. In the SCOP database, proteins with similar domain structures are usually clustered into the same fold hierarchy. Even though manual classification provides reliable results, it is labor intensive. As of May 30th, 2006, 10864 newly-discovered proteins deposited in the Protein Data Bank (PDB) [12] have not been classified in the latest SCOP *v*1.69 release. The number of newly-discovered proteins is increasing continuously.

Recent studies [13,14] apply a consensus scheme to classify the SCOP folds for newly-discovered proteins by intersecting multiple classification results from classical structural alignment algorithms such as DALI [9], Combinatorial Extension (CE) [15] and VAST [16]. These consensus approaches yield higher classification accuracies than each individual method. However, a combination of structural alignment algorithms is computationally expensive. To accelerate the manual classification process of SCOP, there is an urgent need to develop a fast, automated SCOP fold classification system with a reasonably high accuracy. By extending our recent works with the real-time tertiary structure retrieval system, ProteinDBS [17-19], we have already studied an efficient model of association rule (AR) mining to identify relevant structural patterns in proteins for SCOP domain and fold classifications [20]. In this paper, we further develop a non-parametric classifier to conduct the SCOP fold classifications with better accuracy and efficiency. Our contribution is to introduce a real-time classification model, *E-Predict*, that applies the *E_Measure *metric [21] from the Information Retrieval (*IR*) field to assign the known SCOP folds and recognize the novel folds for newly-discovered proteins. In the past, a number of systems have been developed to assign a protein structure to an existing fold or recognize it as a novel fold. For example, DALI [22] uses Z-score of the best structural match to either assign a structure to a known fold (Z>2) or novel fold (Z≤2). Other programs, such as CE [15] and VAST [23] can perform similar tasks. However, the computational effort associated with those methods prevents a user from exploring the protein structure database in real time.

## Results

There are two important tasks for the SCOP fold classifications. 1) *Known SCOP Fold Assignments*: the algorithm assigns newly-discovered protein structures into the known SCOP folds. 2) *Novel SCOP Fold Recognitions*: the algorithm detects whether or not newly-discovered protein structures should be categorized into the novel folds. Given two SCOP database releases *v*_1 _and *v*_2 _(*v*_1 _⊂ *v*_2_), Δv1v2
 MathType@MTEF@5@5@+=feaafiart1ev1aaatCvAUfKttLearuWrP9MDH5MBPbIqV92AaeXatLxBI9gBaebbnrfifHhDYfgasaacH8akY=wiFfYdH8Gipec8Eeeu0xXdbba9frFj0=OqFfea0dXdd9vqai=hGuQ8kuc9pgc9s8qqaq=dirpe0xb9q8qiLsFr0=vr0=vr0dc8meaabaqaciaacaGaaeqabaqabeGadaaakeaacqqHuoardaqhaaWcbaGaemODay3aaSbaaWqaaiabigdaXaqabaaaleaacqWG2bGDdaWgaaadbaGaeGOmaidabeaaaaaaaa@3370@ denotes a set of newly-discovered proteins in *v*_2 _that have not been identified in *v*_1_. The proteins from Δv1v2
 MathType@MTEF@5@5@+=feaafiart1ev1aaatCvAUfKttLearuWrP9MDH5MBPbIqV92AaeXatLxBI9gBaebbnrfifHhDYfgasaacH8akY=wiFfYdH8Gipec8Eeeu0xXdbba9frFj0=OqFfea0dXdd9vqai=hGuQ8kuc9pgc9s8qqaq=dirpe0xb9q8qiLsFr0=vr0=vr0dc8meaabaqaciaacaGaaeqabaqabeGadaaakeaacqqHuoardaqhaaWcbaGaemODay3aaSbaaWqaaiabigdaXaqabaaaleaacqWG2bGDdaWgaaadbaGaeGOmaidabeaaaaaaaa@3370@ will be partitioned into either the known SCOP folds of *v*_1 _(Δv1v2,known
 MathType@MTEF@5@5@+=feaafiart1ev1aaatCvAUfKttLearuWrP9MDH5MBPbIqV92AaeXatLxBI9gBaebbnrfifHhDYfgasaacH8akY=wiFfYdH8Gipec8Eeeu0xXdbba9frFj0=OqFfea0dXdd9vqai=hGuQ8kuc9pgc9s8qqaq=dirpe0xb9q8qiLsFr0=vr0=vr0dc8meaabaqaciaacaGaaeqabaqabeGadaaakeaacqqHuoardaqhaaWcbaGaemODay3aaSbaaWqaaiabigdaXaqabaaaleaacqWG2bGDdaWgaaadbaGaeGOmaidabeaaliabcYcaSiabdUgaRjabd6gaUjabd+gaVjabdEha3jabd6gaUbaaaaa@3B62@), or the novel folds that have not been determined prior to *v*_2 _(Δv1v2,novel
 MathType@MTEF@5@5@+=feaafiart1ev1aaatCvAUfKttLearuWrP9MDH5MBPbIqV92AaeXatLxBI9gBaebbnrfifHhDYfgasaacH8akY=wiFfYdH8Gipec8Eeeu0xXdbba9frFj0=OqFfea0dXdd9vqai=hGuQ8kuc9pgc9s8qqaq=dirpe0xb9q8qiLsFr0=vr0=vr0dc8meaabaqaciaacaGaaeqabaqabeGadaaakeaacqqHuoardaqhaaWcbaGaemODay3aaSbaaWqaaiabigdaXaqabaaaleaacqWG2bGDdaWgaaadbaGaeGOmaidabeaaliabcYcaSiabd6gaUjabd+gaVjabdAha2jabdwgaLjabdYgaSbaaaaa@3B50@), where Δv1v2,known∪Δv1v2,novel=Δv1v2
 MathType@MTEF@5@5@+=feaafiart1ev1aaatCvAUfKttLearuWrP9MDH5MBPbIqV92AaeXatLxBI9gBaebbnrfifHhDYfgasaacH8akY=wiFfYdH8Gipec8Eeeu0xXdbba9frFj0=OqFfea0dXdd9vqai=hGuQ8kuc9pgc9s8qqaq=dirpe0xb9q8qiLsFr0=vr0=vr0dc8meaabaqaciaacaGaaeqabaqabeGadaaakeaacqqHuoardaqhaaWcbaGaemODay3aaSbaaWqaaiabigdaXaqabaaaleaacqWG2bGDdaWgaaadbaGaeGOmaidabeaaliabcYcaSiabdUgaRjabd6gaUjabd+gaVjabdEha3jabd6gaUbaakmaataaabaGaeuiLdq0aa0baaSqaaiabdAha2naaBaaameaacqaIXaqmaeqaaaWcbaGaemODay3aaSbaaWqaaiabikdaYaqabaWccqGGSaalcqWGUbGBcqWGVbWBcqWG2bGDcqWGLbqzcqWGSbaBaaaabeqab0GaeSOkIufakiabg2da9iabfs5aenaaDaaaleaacqWG2bGDdaWgaaadbaGaeGymaedabeaaaSqaaiabdAha2naaBaaameaacqaIYaGmaeqaaaaaaaa@533E@. In our experiments, we measure the classification accuracy for proteins from Δv1v2,known
 MathType@MTEF@5@5@+=feaafiart1ev1aaatCvAUfKttLearuWrP9MDH5MBPbIqV92AaeXatLxBI9gBaebbnrfifHhDYfgasaacH8akY=wiFfYdH8Gipec8Eeeu0xXdbba9frFj0=OqFfea0dXdd9vqai=hGuQ8kuc9pgc9s8qqaq=dirpe0xb9q8qiLsFr0=vr0=vr0dc8meaabaqaciaacaGaaeqabaqabeGadaaakeaacqqHuoardaqhaaWcbaGaemODay3aaSbaaWqaaiabigdaXaqabaaaleaacqWG2bGDdaWgaaadbaGaeGOmaidabeaaliabcYcaSiabdUgaRjabd6gaUjabd+gaVjabdEha3jabd6gaUbaaaaa@3B62@, and then we gauge the accuracy for classifying proteins from Δv1v2,novel
 MathType@MTEF@5@5@+=feaafiart1ev1aaatCvAUfKttLearuWrP9MDH5MBPbIqV92AaeXatLxBI9gBaebbnrfifHhDYfgasaacH8akY=wiFfYdH8Gipec8Eeeu0xXdbba9frFj0=OqFfea0dXdd9vqai=hGuQ8kuc9pgc9s8qqaq=dirpe0xb9q8qiLsFr0=vr0=vr0dc8meaabaqaciaacaGaaeqabaqabeGadaaakeaacqqHuoardaqhaaWcbaGaemODay3aaSbaaWqaaiabigdaXaqabaaaleaacqWG2bGDdaWgaaadbaGaeGOmaidabeaaliabcYcaSiabd6gaUjabd+gaVjabdAha2jabdwgaLjabdYgaSbaaaaa@3B50@. Finally, we report the efficiency of SCOP fold classifications.

### Assigning newly-discovered proteins to the known folds

We conduct three experiments for classifying newly-discovered proteins into the known folds. The first experiment compares our classification model, *E-Predict*, with several methods reported in a recent work [13] such as CE, DALI, VAST and CBOOST. Our test data shown in Table 1 is the same test set used in their work, which has proteins with average sequence identities equal to 16.88% and average sequence similarities equal to 20.76% by conducting all against all pairwise alignments using *EMBOSS-Align *[24] algorithm. The same ground truth data with their work includes proteins from the entire SCOP *v*l.59 release. To evaluate the accuracy, we use a general metric, *Correct Classification Rate *(*CCR*), which is defined as follows:

CCR=The number of correctly classified proteinsThe total number of test proteins     (1)
 MathType@MTEF@5@5@+=feaafiart1ev1aaatCvAUfKttLearuWrP9MDH5MBPbIqV92AaeXatLxBI9gBaebbnrfifHhDYfgasaacH8akY=wiFfYdH8Gipec8Eeeu0xXdbba9frFj0=OqFfea0dXdd9vqai=hGuQ8kuc9pgc9s8qqaq=dirpe0xb9q8qiLsFr0=vr0=vr0dc8meaabaqaciaacaGaaeqabaqabeGadaaakeaacqWGdbWqcqWGdbWqcqWGsbGucqGH9aqpdaWcaaqaaiabdsfaujabdIgaOjabdwgaLjabbccaGiabd6gaUjabdwha1jabd2gaTjabdkgaIjabdwgaLjabdkhaYjabbccaGiabd+gaVjabdAgaMjabbccaGiabdogaJjabd+gaVjabdkhaYjabdkhaYjabdwgaLjabdogaJjabdsha0jabdYgaSjabdMha5jabbccaGiabdogaJjabdYgaSjabdggaHjabdohaZjabdohaZjabdMgaPjabdAgaMjabdMgaPjabdwgaLjabdsgaKjabbccaGiabdchaWjabdkhaYjabd+gaVjabdsha0jabdwgaLjabdMgaPjabd6gaUjabdohaZbqaaiabdsfaujabdIgaOjabdwgaLjabbccaGiabdsha0jabd+gaVjabdsha0jabdggaHHqaciab=XgaSjabbccaGiab=5gaUjab=vha1jabd2gaTjabdkgaIjabdwgaLjabdkhaYjabbccaGiabd+gaVjabdAgaMjabbccaGiabdsha0jabdwgaLjabdohaZjabdsha0jabbccaGiabdchaWjabdkhaYjabd+gaVjabdsha0jabdwgaLjabdMgaPjabd6gaUjabdohaZbaacaWLjaGaaCzcamaabmGabaGaeGymaedacaGLOaGaayzkaaaaaa@9753@

**Figure 1:**
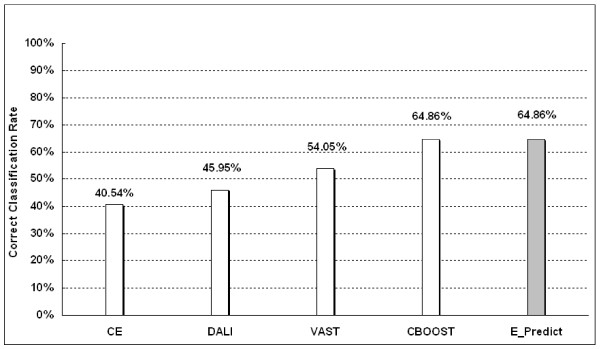
The *Correct Classification Rate *of assigning the known folds for test proteins in Table 1.

Figure 1 shows that *E-Predict *outperforms DALI, CE, and VAST, exhibiting an accuracy of 64.86%. Can *et al*. [13] have proposed a method, named CBOOST, which utilizes a decision tree to integrate DALI, CE, and VAST, achieving the same accuracy of 64.86%. It is worth mentioning that the computationally expensive structural alignment algorithms of CBOOST may not be able to efficiently classify a large number of newly-discovered proteins generated from on-going, high-throughput structure determination projects.

**Figure 2:**
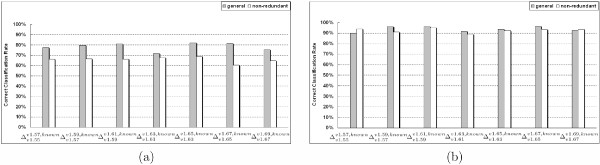
The *Correct Classification Rate *of assigning the known folds for various SCOP releases using *E-Predict *on (a) general and non-redundant test set in Δv1v2,known
 MathType@MTEF@5@5@+=feaafiart1ev1aaatCvAUfKttLearuWrP9MDH5MBPbIqV92AaeXatLxBI9gBaebbnrfifHhDYfgasaacH8akY=wiFfYdH8Gipec8Eeeu0xXdbba9frFj0=OqFfea0dXdd9vqai=hGuQ8kuc9pgc9s8qqaq=dirpe0xb9q8qiLsFr0=vr0=vr0dc8meaabaqaciaacaGaaeqabaqabeGadaaakeaacqqHuoardaqhaaWcbaGaemODay3aaSbaaWqaaiabigdaXaqabaaaleaacqWG2bGDdaWgaaadbaGaeGOmaidabeaaliabcYcaSiabdUgaRjabd6gaUjabd+gaVjabdEha3jabd6gaUbaaaaa@3B62@ which are selected from the known SCOP folds of *v*_2 _with at least one protein chain in *v*_1 _(Table 2) (b) general and non-redundant test set in Δv1v2,known
 MathType@MTEF@5@5@+=feaafiart1ev1aaatCvAUfKttLearuWrP9MDH5MBPbIqV92AaeXatLxBI9gBaebbnrfifHhDYfgasaacH8akY=wiFfYdH8Gipec8Eeeu0xXdbba9frFj0=OqFfea0dXdd9vqai=hGuQ8kuc9pgc9s8qqaq=dirpe0xb9q8qiLsFr0=vr0=vr0dc8meaabaqaciaacaGaaeqabaqabeGadaaakeaacqqHuoardaqhaaWcbaGaemODay3aaSbaaWqaaiabigdaXaqabaaaleaacqWG2bGDdaWgaaadbaGaeGOmaidabeaaliabcYcaSiabdUgaRjabd6gaUjabd+gaVjabdEha3jabd6gaUbaaaaa@3B62@ which are selected from the known SCOP folds of *v*_2 _with at least 10 protein chains in *v*_1 _(Table 3).

**Figure 3:**
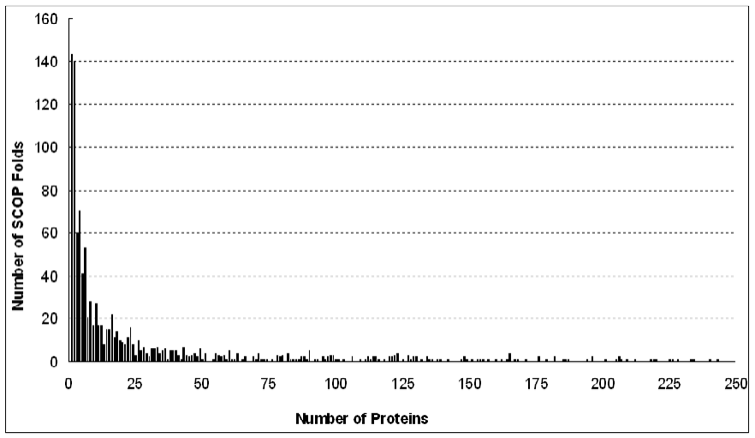
The amount of proteins in the folds against the number of SCOP folds in the SCOP *v*1.69 release.

The second experiment exhaustively evaluates the accuracy of *E-Predict *on several general test sets from Δv1.55,generalv1.57,known
 MathType@MTEF@5@5@+=feaafiart1ev1aaatCvAUfKttLearuWrP9MDH5MBPbIqV92AaeXatLxBI9gBaebbnrfifHhDYfgasaacH8akY=wiFfYdH8Gipec8Eeeu0xXdbba9frFj0=OqFfea0dXdd9vqai=hGuQ8kuc9pgc9s8qqaq=dirpe0xb9q8qiLsFr0=vr0=vr0dc8meaabaqaciaacaGaaeqabaqabeGadaaakeaacqqHuoardaqhaaWcbaGaemODayNaeGymaeJaeiOla4IaeGynauJaeGynauJaeiilaWIaem4zaCMaemyzauMaemOBa4MaemyzauMaemOCaiNaemyyaeMaemiBaWgabaGaemODayNaeGymaeJaeiOla4IaeGynauJaeG4naCJaeiilaWIaem4AaSMaemOBa4Maem4Ba8Maem4DaCNaemOBa4gaaaaa@4AF7@ to Δv1.67,generalv1.69,known
 MathType@MTEF@5@5@+=feaafiart1ev1aaatCvAUfKttLearuWrP9MDH5MBPbIqV92AaeXatLxBI9gBaebbnrfifHhDYfgasaacH8akY=wiFfYdH8Gipec8Eeeu0xXdbba9frFj0=OqFfea0dXdd9vqai=hGuQ8kuc9pgc9s8qqaq=dirpe0xb9q8qiLsFr0=vr0=vr0dc8meaabaqaciaacaGaaeqabaqabeGadaaakeaacqqHuoardaqhaaWcbaGaemODayNaeGymaeJaeiOla4IaeGOnayJaeG4naCJaeiilaWIaem4zaCMaemyzauMaemOBa4MaemyzauMaemOCaiNaemyyaeMaemiBaWgabaGaemODayNaeGymaeJaeiOla4IaeGOnayJaeGyoaKJaeiilaWIaem4AaSMaemOBa4Maem4Ba8Maem4DaCNaemOBa4gaaaaa@4B03@. In Table 2 and Table 3, our test proteins in Δv1v2,known
 MathType@MTEF@5@5@+=feaafiart1ev1aaatCvAUfKttLearuWrP9MDH5MBPbIqV92AaeXatLxBI9gBaebbnrfifHhDYfgasaacH8akY=wiFfYdH8Gipec8Eeeu0xXdbba9frFj0=OqFfea0dXdd9vqai=hGuQ8kuc9pgc9s8qqaq=dirpe0xb9q8qiLsFr0=vr0=vr0dc8meaabaqaciaacaGaaeqabaqabeGadaaakeaacqqHuoardaqhaaWcbaGaemODay3aaSbaaWqaaiabigdaXaqabaaaleaacqWG2bGDdaWgaaadbaGaeGOmaidabeaaliabcYcaSiabdUgaRjabd6gaUjabd+gaVjabdEha3jabd6gaUbaaaaa@3B62@ are selected from the known SCOP folds of *v*_2_, which also maintain at least one protein chain and 10 proteins in *v*_1_, respectively. Figure 2(a) shows that *E-Predict *achieves 72% to 82% classification accuracies for the general test sets of seven SCOP releases. According to Figure 3, there exists a large number of SCOP folds with small sizes. When a newly-discovered protein belongs to a small-size fold, there is a limited amount of ground truth data available. In machine learning, classifiers usually require sufficient ground truth data to guarantee the accuracy. Figure 2(b) demonstrates that *E-Predict *is able to achieve much higher accuracies, 90% to 96%, for the general test sets of seven SCOP releases with more than 10 ground truth proteins. In the future, when newly-discovered protein structures are categorized into those small-size SCOP folds, the accuracy of *E-Predict *could be further improved.

The third experiment evaluates the accuracy of *E-Predict *on *non-redundant *test sets, which are obtained from randomly sampling one protein chain among each SCOP superfamily. In Table 2 and Table 3, a *non-redundant *test set Δv1,non−redundantv2,known
 MathType@MTEF@5@5@+=feaafiart1ev1aaatCvAUfKttLearuWrP9MDH5MBPbIqV92AaeXatLxBI9gBaebbnrfifHhDYfgasaacH8akY=wiFfYdH8Gipec8Eeeu0xXdbba9frFj0=OqFfea0dXdd9vqai=hGuQ8kuc9pgc9s8qqaq=dirpe0xb9q8qiLsFr0=vr0=vr0dc8meaabaqaciaacaGaaeqabaqabeGadaaakeaacqqHuoardaqhaaWcbaGaemODay3aaSbaaWqaaiabigdaXaqabaWccqGGSaalcqWGUbGBcqWGVbWBcqWGUbGBcqGHsislcqWGYbGCcqWGLbqzcqWGKbazcqWG1bqDcqWGUbGBcqWGKbazcqWGHbqycqWGUbGBcqWG0baDaeaacqWG2bGDdaWgaaadbaGaeGOmaidabeaaliabcYcaSiabdUgaRjabd6gaUjabd+gaVjabdEha3jabd6gaUbaaaaa@4DBB@ is defined by randomly selecting one protein from each SCOP superfamily of the general test set Δv1,generalv2,known
 MathType@MTEF@5@5@+=feaafiart1ev1aaatCvAUfKttLearuWrP9MDH5MBPbIqV92AaeXatLxBI9gBaebbnrfifHhDYfgasaacH8akY=wiFfYdH8Gipec8Eeeu0xXdbba9frFj0=OqFfea0dXdd9vqai=hGuQ8kuc9pgc9s8qqaq=dirpe0xb9q8qiLsFr0=vr0=vr0dc8meaabaqaciaacaGaaeqabaqabeGadaaakeaacqqHuoardaqhaaWcbaGaemODay3aaSbaaWqaaiabigdaXaqabaWccqGGSaalcqWGNbWzcqWGLbqzcqWGUbGBcqWGLbqzcqWGYbGCcqWGHbqycqWGSbaBaeaacqWG2bGDdaWgaaadbaGaeGOmaidabeaaliabcYcaSiabdUgaRjabd6gaUjabd+gaVjabdEha3jabd6gaUbaaaaa@45BD@. According to SCOP [11], proteins between two different SCOP superfamilies have low sequence similarities, which suggest that test proteins in our *non-redundant *sets should maintain low sequence similarities. Table 4 measures the degree of sequence redundancy for 10 pairs of proteins, which are randomly sampled from the *non-redundant *set Δv1.67,non−redundantv1.69,known
 MathType@MTEF@5@5@+=feaafiart1ev1aaatCvAUfKttLearuWrP9MDH5MBPbIqV92AaeXatLxBI9gBaebbnrfifHhDYfgasaacH8akY=wiFfYdH8Gipec8Eeeu0xXdbba9frFj0=OqFfea0dXdd9vqai=hGuQ8kuc9pgc9s8qqaq=dirpe0xb9q8qiLsFr0=vr0=vr0dc8meaabaqaciaacaGaaeqabaqabeGadaaakeaacqqHuoardaqhaaWcbaGaemODayNaeGymaeJaeiOla4IaeGOnayJaeG4naCJaeiilaWIaemOBa4Maem4Ba8MaemOBa4MaeyOeI0IaemOCaiNaemyzauMaemizaqMaemyDauNaemOBa4MaemizaqMaemyyaeMaemOBa4MaemiDaqhabaGaemODayNaeGymaeJaeiOla4IaeGOnayJaeGyoaKJaeiilaWIaem4AaSMaemOBa4Maem4Ba8Maem4DaCNaemOBa4gaaaaa@5301@ with the average sequence identity and sequence similarity equal to 12.55% and 21.17%, respectively. In addition, the experiment using the *non-redundant *test sets avoids the case that some folds in the general test sets predominate the classification accuracy with relatively more test proteins. For example, there are 900 out of 1000 test proteins in a general test from the same SCOP fold *f*_1_. The quantity of this fold may affect the accuracy significantly when a majority of these 900 proteins are correctly classified. In Figure 2(a), *E-Predict *presents a reduction of accuracies on several sets of *non-redundant *proteins in comparison with the general test sets in Table 2, which includes small-size folds. This gap demonstrates that the impact of some SCOP folds with outnumbered proteins in the general test sets improves the overall accuracy. Figure 2(b) shows that *E-Predict *exhibits similar accuracies on seven sets of the *non-redundant *proteins in comparison with the general test sets in Table 3, which have at least 10 ground truth proteins. This suggests that with a sufficient amount of ground truth data *non-redundant *proteins can still be classified with a reasonably high accuracy.

### Recognizing the novel folds for newly-discovered proteins

**Figure 4:**
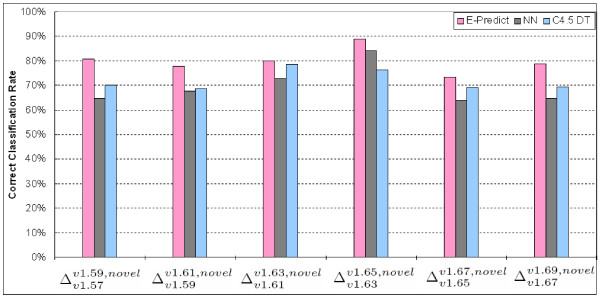
The *Correct Classification Rates *of recognizing the novel SCOP folds for proteins in various SCOP releases.

We measure the accuracies of classifying six sets of proteins with the novel folds from Δv1.57v1.59,novel
 MathType@MTEF@5@5@+=feaafiart1ev1aaatCvAUfKttLearuWrP9MDH5MBPbIqV92AaeXatLxBI9gBaebbnrfifHhDYfgasaacH8akY=wiFfYdH8Gipec8Eeeu0xXdbba9frFj0=OqFfea0dXdd9vqai=hGuQ8kuc9pgc9s8qqaq=dirpe0xb9q8qiLsFr0=vr0=vr0dc8meaabaqaciaacaGaaeqabaqabeGadaaakeaacqqHuoardaqhaaWcbaGaemODayNaeGymaeJaeiOla4IaeGynauJaeG4naCdabaGaemODayNaeGymaeJaeiOla4IaeGynauJaeGyoaKJaeiilaWIaemOBa4Maem4Ba8MaemODayNaemyzauMaemiBaWgaaaaa@4092@ to Δv1.67v1.69,novel
 MathType@MTEF@5@5@+=feaafiart1ev1aaatCvAUfKttLearuWrP9MDH5MBPbIqV92AaeXatLxBI9gBaebbnrfifHhDYfgasaacH8akY=wiFfYdH8Gipec8Eeeu0xXdbba9frFj0=OqFfea0dXdd9vqai=hGuQ8kuc9pgc9s8qqaq=dirpe0xb9q8qiLsFr0=vr0=vr0dc8meaabaqaciaacaGaaeqabaqabeGadaaakeaacqqHuoardaqhaaWcbaGaemODayNaeGymaeJaeiOla4IaeGOnayJaeG4naCdabaGaemODayNaeGymaeJaeiOla4IaeGOnayJaeGyoaKJaeiilaWIaemOBa4Maem4Ba8MaemODayNaemyzauMaemiBaWgaaaaa@4096@, which are listed in Table 2. We accumulate labeled proteins from the prior SCOP releases to obtain more ground truth data. For example, when an experiment is conducted with test proteins from Δv1.67v1.69,novel
 MathType@MTEF@5@5@+=feaafiart1ev1aaatCvAUfKttLearuWrP9MDH5MBPbIqV92AaeXatLxBI9gBaebbnrfifHhDYfgasaacH8akY=wiFfYdH8Gipec8Eeeu0xXdbba9frFj0=OqFfea0dXdd9vqai=hGuQ8kuc9pgc9s8qqaq=dirpe0xb9q8qiLsFr0=vr0=vr0dc8meaabaqaciaacaGaaeqabaqabeGadaaakeaacqqHuoardaqhaaWcbaGaemODayNaeGymaeJaeiOla4IaeGOnayJaeG4naCdabaGaemODayNaeGymaeJaeiOla4IaeGOnayJaeGyoaKJaeiilaWIaemOBa4Maem4Ba8MaemODayNaemyzauMaemiBaWgaaaaa@4096@, our ground truth data is composed of new proteins from Δv1.55v1.67
 MathType@MTEF@5@5@+=feaafiart1ev1aaatCvAUfKttLearuWrP9MDH5MBPbIqV92AaeXatLxBI9gBaebbnrfifHhDYfgasaacH8akY=wiFfYdH8Gipec8Eeeu0xXdbba9frFj0=OqFfea0dXdd9vqai=hGuQ8kuc9pgc9s8qqaq=dirpe0xb9q8qiLsFr0=vr0=vr0dc8meaabaqaciaacaGaaeqabaqabeGadaaakeaacqqHuoardaqhaaWcbaGaemODayNaeGymaeJaeiOla4IaeGynauJaeGynaudabaGaemODayNaeGymaeJaeiOla4IaeGOnayJaeG4naCdaaaaa@38B7@. We compare our *E-Predict *algorithm with two prevalent classification methods, *Nearest Neighbor *search (NN) [25] and *C4.5 Decision Tree *(*DT*) [26]. Figure 4 presents a plot of *CCR *against six test sets Δv1.57v1.59,novel
 MathType@MTEF@5@5@+=feaafiart1ev1aaatCvAUfKttLearuWrP9MDH5MBPbIqV92AaeXatLxBI9gBaebbnrfifHhDYfgasaacH8akY=wiFfYdH8Gipec8Eeeu0xXdbba9frFj0=OqFfea0dXdd9vqai=hGuQ8kuc9pgc9s8qqaq=dirpe0xb9q8qiLsFr0=vr0=vr0dc8meaabaqaciaacaGaaeqabaqabeGadaaakeaacqqHuoardaqhaaWcbaGaemODayNaeGymaeJaeiOla4IaeGynauJaeG4naCdabaGaemODayNaeGymaeJaeiOla4IaeGynauJaeGyoaKJaeiilaWIaemOBa4Maem4Ba8MaemODayNaemyzauMaemiBaWgaaaaa@4092@ to Δv1.67v1.69,novel
 MathType@MTEF@5@5@+=feaafiart1ev1aaatCvAUfKttLearuWrP9MDH5MBPbIqV92AaeXatLxBI9gBaebbnrfifHhDYfgasaacH8akY=wiFfYdH8Gipec8Eeeu0xXdbba9frFj0=OqFfea0dXdd9vqai=hGuQ8kuc9pgc9s8qqaq=dirpe0xb9q8qiLsFr0=vr0=vr0dc8meaabaqaciaacaGaaeqabaqabeGadaaakeaacqqHuoardaqhaaWcbaGaemODayNaeGymaeJaeiOla4IaeGOnayJaeG4naCdabaGaemODayNaeGymaeJaeiOla4IaeGOnayJaeGyoaKJaeiilaWIaemOBa4Maem4Ba8MaemODayNaemyzauMaemiBaWgaaaaa@4096@, which are listed in Table 2. From computational results, *E-Predict *outperforms NN and *C4.5 DT*. There is a noticeable reduction in accuracy when classifying proteins in Δv1.65v1.67,novel
 MathType@MTEF@5@5@+=feaafiart1ev1aaatCvAUfKttLearuWrP9MDH5MBPbIqV92AaeXatLxBI9gBaebbnrfifHhDYfgasaacH8akY=wiFfYdH8Gipec8Eeeu0xXdbba9frFj0=OqFfea0dXdd9vqai=hGuQ8kuc9pgc9s8qqaq=dirpe0xb9q8qiLsFr0=vr0=vr0dc8meaabaqaciaacaGaaeqabaqabeGadaaakeaacqqHuoardaqhaaWcbaGaemODayNaeGymaeJaeiOla4IaeGOnayJaeGynaudabaGaemODayNaeGymaeJaeiOla4IaeGOnayJaeG4naCJaeiilaWIaemOBa4Maem4Ba8MaemODayNaemyzauMaemiBaWgaaaaa@408E@. This is probably because the test set, Δv1.65v1.67,novel
 MathType@MTEF@5@5@+=feaafiart1ev1aaatCvAUfKttLearuWrP9MDH5MBPbIqV92AaeXatLxBI9gBaebbnrfifHhDYfgasaacH8akY=wiFfYdH8Gipec8Eeeu0xXdbba9frFj0=OqFfea0dXdd9vqai=hGuQ8kuc9pgc9s8qqaq=dirpe0xb9q8qiLsFr0=vr0=vr0dc8meaabaqaciaacaGaaeqabaqabeGadaaakeaacqqHuoardaqhaaWcbaGaemODayNaeGymaeJaeiOla4IaeGOnayJaeGynaudabaGaemODayNaeGymaeJaeiOla4IaeGOnayJaeG4naCJaeiilaWIaemOBa4Maem4Ba8MaemODayNaemyzauMaemiBaWgaaaaa@408E@, is harder to be correctly predicted than the other sets. To address the issue that accuracies may be biased by particular new structures, we conduct 10 fold cross validation that sequentially selects 10% of ground truth data from Δv1.55v1.69
 MathType@MTEF@5@5@+=feaafiart1ev1aaatCvAUfKttLearuWrP9MDH5MBPbIqV92AaeXatLxBI9gBaebbnrfifHhDYfgasaacH8akY=wiFfYdH8Gipec8Eeeu0xXdbba9frFj0=OqFfea0dXdd9vqai=hGuQ8kuc9pgc9s8qqaq=dirpe0xb9q8qiLsFr0=vr0=vr0dc8meaabaqaciaacaGaaeqabaqabeGadaaakeaacqqHuoardaqhaaWcbaGaemODayNaeGymaeJaeiOla4IaeGynauJaeGynaudabaGaemODayNaeGymaeJaeiOla4IaeGOnayJaeGyoaKdaaaaa@38BB@ as a test set and the rest of 90% of ground truth data as a training set for 10 times. In the 10 fold experiment, our approach achieves 89.27% accuracy of the novel fold recognitions.

### Efficiency

**Figure 5:**
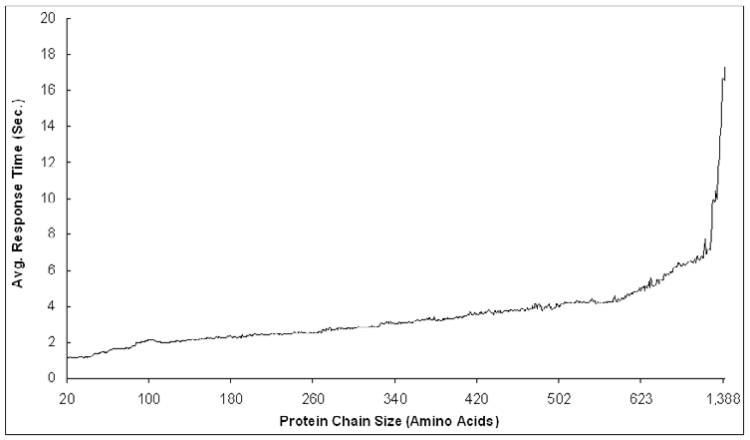
The protein chain sizes against the average response time of classifying test proteins.

For efficiency, we measure the average response time of the entire classification process, including feature extraction, nearest neighbor search on an M-tree [27], and the computation of the SCOP folds by the *E-Predict *algorithm. The classification process performs *one-against-all *structural comparisons by scanning the entire SCOP database. Our system runs on a Fedora-Core Linux system with Dual Xeon IV 2.4 GHz processors and 2 GB RAM. A large-scale test set is chosen from the SCOP *v*l.69 release with 51911 protein chains which have more than 20 amino acids. Figure 5 shows the average response time of fold classifications for various protein chain sizes. When the protein size increases, the *E-Predict *algorithm demands more computational resources to extract features from larger distance matrices. When the protein chain size reaches a certain threshold, the Linux system may swap huge distance matrices into the virtual memory resulting in a significant *I/O *time. This effect is reflected in Figure 5 with long computation times for the protein chain size larger than 1099 amino acids, where more memory is required to prevent page swapping. On average, classifying a newly-discovered protein to a SCOP fold takes 3.5 seconds. In our test set, the longest protein chain, comprised of 1409 amino acids, completes the classification process in 17.4 seconds.

## Discussion

Our approach yields better accuracy and efficiency compared to the structure alignment algorithms. The accuracy is achieved by analyzing the ranked SCOP folds of a nearest neighbors search using the *E-Predict *algorithm. In addition, efficiency results from using an M-tree [27] for fast nearest neighbor searches. In the following subsections, we compare our performance with the structural alignment algorithms in terms of efficiency and accuracy.

### Performance in efficiency

Since structural alignment algorithms usually apply dynamic programming techniques to align each pair of amino acids in two proteins, they demand a huge amount of computational resources. Instead of aligning amino acids, our *E-Predict *model transforms relevant protein structure information into high-level features, and similar protein structures are then retrieved from a high-dimensional feature space by a nearest neighbors search in the M-tree. Our approach is able to return the classification result in seconds. Since performing the structural alignment algorithms with multiple pairwise alignments of a newly-discovered structure against the known protein structures from the SCOP database is known to be computationally expensive [10], the response times for the structural alignment algorithms are not plotted in Figure 5.

### The accuracy of assigning newly-discovered proteins to the known folds

For the assignment of proteins to the known SCOP folds, the *E-Predict *algorithm mainly contributes to the accuracy. Traditional structural alignment methods usually apply heuristics to reduce computational efforts of aligning a large combination of amino acids in two proteins. Different heuristics could return diverse results from the same set of proteins since these algorithms might be trapped in local optimal solutions. Even though a consensus method that combines classification results of multiple structural alignment algorithms outperforms each individual structural alignment approach [13], it is computationally expensive. Instead of performing structural alignments, our model maps both known proteins from the SCOP database and newly-discovered protein structures into 33-D feature vectors. With a nearest neighbor search for a newly-discovered structure *t *in the high-dimensional feature space, there may exist multiple candidate folds, which are associated with nearest neighbor proteins in the vicinity of *t*. One way to assign a SCOP fold to *t *is to choose the fold of the nearest neighbor protein in the high-dimensional feature space. Since it is possible that hundreds of folds are partially overlapped in the high-dimensional feature space, the nearest neighbor of *t *may be an outlier that deviates from the majority of proteins in its fold. To avoid selecting an outlier, we apply the *E*_*Measure *metric that considers the ranks of at least two nearest neighbor proteins for each fold. The algorithm rewards a SCOP fold in which proteins are highly ranked and penalizes a fold with proteins in the lower ranks. Hence, when the SCOP fold includes only a single highly ranked protein with the other proteins from this fold ranked much lower, the algorithm is able to avoid assigning this fold to *t *based on the penalty of low ranking. From computational results, *E*_*Measure *has a vital impact on the classification accuracy.

### Misclassifications of assigning newly-discovered proteins to the known folds

**Figure 6:**
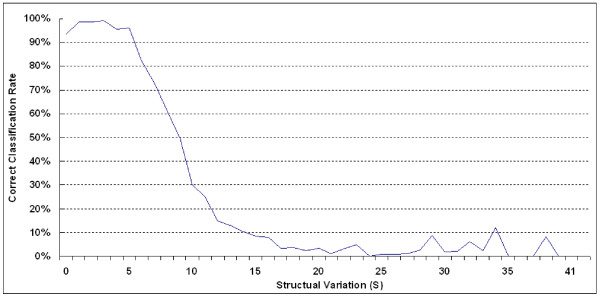
*Correct Classification Rates *of classifying test proteins against structural variation values.

Within the framework of ProteinDBS [17-19], our model, *E-Predict*, transforms a 3-D protein structure into a 33-D feature vector that represents the geometric properties of folded proteins. Applying these features to measure the structural similarity of proteins, *E-Predict *outperforms several classification methods that apply the structural alignment algorithm using the test set in Table 1. *E-Predict *also yields reasonably high accuracy for several test sets in Table 3 with sufficient ground truth data. However, misclassifications still exist. The limited amount of 33-D ground truth data available for training contributes to the classification errors. As more ground truth data becomes available in small-size SCOP folds, a higher classification accuracy is expected. The second reason for misclassifications is due to the overlapping of folds in the high-dimensional feature space. To further separate overlapping folds, our system needs more relevant features to detect the protein 3-D folding with sufficient discriminating power. Another possible reason for misclassifications is that SCOP may categorize a partial segment of a PDB protein chain (substructure) into a domain. Since our approach measures the global similarity of distance matrices for classification, users need to submit the portion of the protein chain identified in the SCOP domain to ensure a correct classification. In Figure 6, we measure the correlation between the classification accuracy and a structure variation value, *S*, for a query protein *t *and the best matched protein of *t *in our classified SCOP fold. For a pair of proteins (*p*_1_, *p*_2_), the structural variation *S *is defined as follows:

S(p1,p2)=RMSD/(NANp1+Np2),     (2)
 MathType@MTEF@5@5@+=feaafiart1ev1aaatCvAUfKttLearuWrP9MDH5MBPbIqV92AaeXatLxBI9gBaebbnrfifHhDYfgasaacH8akY=wiFfYdH8Gipec8Eeeu0xXdbba9frFj0=OqFfea0dXdd9vqai=hGuQ8kuc9pgc9s8qqaq=dirpe0xb9q8qiLsFr0=vr0=vr0dc8meaabaqaciaacaGaaeqabaqabeGadaaakeaacqWGtbWucqGGOaakcqWGWbaCdaWgaaWcbaGaeGymaedabeaakiabcYcaSiabdchaWnaaBaaaleaacqaIYaGmaeqaaOGaeiykaKIaeyypa0JaemOuaiLaemyta0Kaem4uamLaemiraqKaei4la8IaeiikaGYaaSaaaeaacqWGobGtdaWgaaWcbaGaemyqaeeabeaaaOqaaiabd6eaonaaBaaaleaacqWGWbaCdaWgaaadbaGaeGymaedabeaaaSqabaGccqGHRaWkcqWGobGtdaWgaaWcbaGaemiCaa3aaSbaaWqaaiabikdaYaqabaaaleqaaaaakiabcMcaPiabcYcaSiaaxMaacaWLjaWaaeWaceaacqaIYaGmaiaawIcacaGLPaaaaaa@4D8E@

where *RMSD *means the root mean square deviation of aligned segments, and *N*_*A *_denotes the number of amino acids in the aligned segments of two proteins. *N*_*p*1 _and *N*_*p*2 _represent the number of amino acid residues in *p*_1 _and *p*_2_, respectively. These measurements are computed using SARF [28]. The smaller *S *value can be interpreted as a better structural match for two proteins *p*_1 _and *p*_2_. Two proteins that have a high structural similarity can usually be superimposed with longer aligned residue segments and a small *RMSD *value, resulting in a small *S *value. For example, the SARF algorithm aligns a query protein *t *with 100 amino acids and its best matched protein *p*_1 _with 100 amino acids and returns structurally similar segments with 90 amino acid residues and 0.3 *Å *of *RMSD*. Their structure variation value *S *is computed as 0.3/(90100+100
 MathType@MTEF@5@5@+=feaafiart1ev1aaatCvAUfKttLearuWrP9MDH5MBPbIqV92AaeXatLxBI9gBaebbnrfifHhDYfgasaacH8akY=wiFfYdH8Gipec8Eeeu0xXdbba9frFj0=OqFfea0dXdd9vqai=hGuQ8kuc9pgc9s8qqaq=dirpe0xb9q8qiLsFr0=vr0=vr0dc8meaabaqaciaacaGaaeqabaqabeGadaaakeaadaWcaaqaaiabiMda5iabicdaWaqaaiabigdaXiabicdaWiabicdaWiabgUcaRiabigdaXiabicdaWiabicdaWaaaaaa@3524@) = 0.67. When *S *is smaller than 6, we expect the *E-Predict *algorithm to maintain above 90% classification accuracy. This statistic is obtained from the classification of 41262 testing proteins.

### The accuracy of recognizing the novel folds for newly-discovered proteins

Since no protein has been labeled with the novel folds in our 33-D ground truth data, the novel fold recognition becomes a challenging problem. To address this issue, we introduce three features: *E*_*Measure *evaluation score, structural variation value, and Euclidean distance measurement. These features measure structural similarity between a newly-discovered protein and the nearest neighbor protein in a candidate known fold suggested by the *E-Predict *algorithm. Then, our method applies the *E-Predict *algorithm as a classifier to identify meaningful patterns from ground truth data, which has been obtained by the aggregation of proteins in several prior SCOP releases. Computational results show that using these three features benefits the classification accuracy.

### Misclassifications of recognizing the novel folds for newly-discovered proteins

To recognize the novel folds for newly-discovered protein structures, our classification model exploits three relevant features. With the assumption that protein structures in the novel folds usually present low structural similarities to proteins in the known folds, a high *E*_*Measure *evaluation score, a high Euclidean distance, and a high structural variation value are expected for newly-discovered protein structures from the novel folds. Due to noise in ground truth data and imperfect features, a few proteins in the novel folds may have a low structural variation value, a low *E*_*Measure *score, or a low Euclidean distance measurement. Even though our approach presents an improved accuracy over NN and *C4.5 DT*, there is still a need to discover more relevant features for better recognition performance.

## Conclusion

We have developed an automatic SCOP fold classification system that is able to assign the known SCOP folds and recognize the novel folds for newly-discovered proteins. For the known fold assignments, the algorithm transforms protein structures into 33-D feature vectors and constructs an M-tree to index these feature vectors for fast retrievals. The *E-Predict *algorithm is then applied to classify newly-discovered proteins in the known SCOP folds. For the novel fold recognitions, the algorithm utilizes three relevant features that are related to structural similarity of proteins. From the computational results, our method outperforms several structural alignment algorithms such as DALI, CE and VAST, achieving reasonably high classification accuracy and efficiency. This research can help accelerate the classification process of the SCOP database and benefit the biomedical research community to further study biochemical functions of proteins with similar 3-D structures.

## Methods

Our classification model, *E-Predict*, contains three primary functions. First, *E-Predict *assigns newly-discovered protein structures to the known SCOP folds. Second, *E-Predict *recognizes the novel folds for newly-discovered protein structures. Third, *E-Predict *indexes the high-dimensional protein data for a fast nearest neighbors search.

### Assigning newly-discovered proteins to the known folds

**Figure 7:**
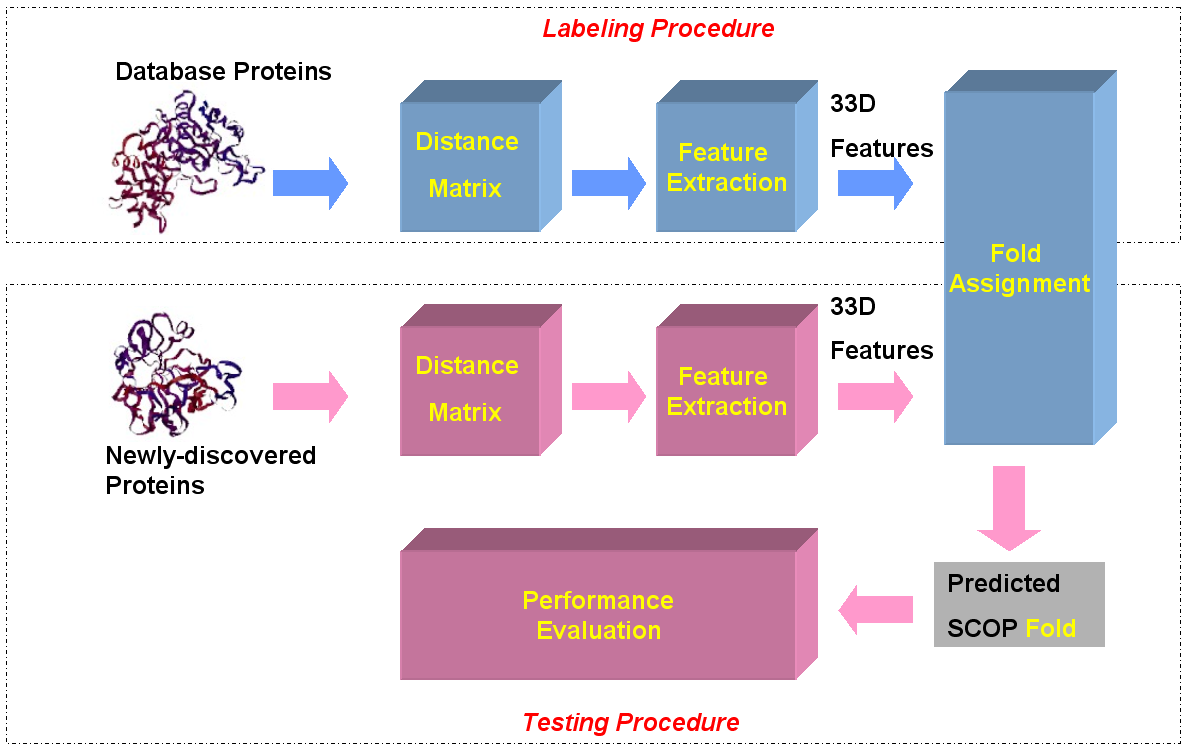
*E-Predict *model for assigning newly-discovered proteins to the known folds.

According to the SCOP hierarchical setting, proteins that share similar secondary structure arrangements are usually classified in the *fold *level [11]. The entire process of assigning newly-discovered proteins to the known folds is shown in Figure 7. The labeling procedure transforms protein structures from the SCOP database into 33-D feature vectors, which are labeled with their corresponding SCOP folds. These labeled proteins are then used as our ground truth data. The testing procedure converts newly-discovered proteins into feature vectors and submits these unlabeled vectors into a classifier to obtain possible SCOP fold assignments. In the following, we discuss several components of the entire process such as distance matrix generation, feature extraction, and classifier design.

#### Mapping 3-D backbone structures into 2-D distance matrices

**Figure 8:**
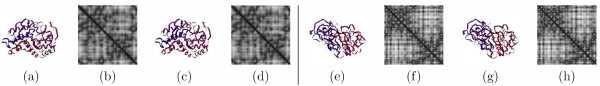
The 3-D backbone structures and distance matrices of four protein chains, which are selected from the SCOP folds: (1)*Heme-dependent peroxidases: *1*kta*_*A*(a-b), 1*ekv*_*A*(c-d), (2)*Acid proteases : *1*lee*_*A*(e-f), 1*lf*2_*A*(g-h).

Proteins are polypeptide chains, which are chained by 20 types of amino acids. Instead of considering the side chains of amino acids, many computational biology papers [6,9,15] use the *C*_*α *_atom to describe each amino acid. In our model, the *C*_*α *_backbone of the *k*^*th *^protein chain with *n *amino acids can be represented by a set of vectors, Ω^*k *^= {Cαk→,1,Cαk→,2,...,Cαk→,n
 MathType@MTEF@5@5@+=feaafiart1ev1aaatCvAUfKttLearuWrP9MDH5MBPbIqV92AaeXatLxBI9gBaebbnrfifHhDYfgasaacH8akY=wiFfYdH8Gipec8Eeeu0xXdbba9frFj0=OqFfea0dXdd9vqai=hGuQ8kuc9pgc9s8qqaq=dirpe0xb9q8qiLsFr0=vr0=vr0dc8meaabaqaciaacaGaaeqabaqabeGadaaakeaacqWGdbWqdaqhaaWcbaacciGae8xSdegabaGafm4AaSMbaSaacqGGSaalcqaIXaqmaaGccqGGSaalcqWGdbWqdaqhaaWcbaGae8xSdegabaGafm4AaSMbaSaacqGGSaalcqaIYaGmaaGccqGGSaalcqGGUaGlcqGGUaGlcqGGUaGlcqGGSaalcqWGdbWqdaqhaaWcbaGae8xSdegabaGafm4AaSMbaSaacqGGSaalcqWGUbGBaaaaaa@44D4@}, where Cαk→,i
 MathType@MTEF@5@5@+=feaafiart1ev1aaatCvAUfKttLearuWrP9MDH5MBPbIqV92AaeXatLxBI9gBaebbnrfifHhDYfgasaacH8akY=wiFfYdH8Gipec8Eeeu0xXdbba9frFj0=OqFfea0dXdd9vqai=hGuQ8kuc9pgc9s8qqaq=dirpe0xb9q8qiLsFr0=vr0=vr0dc8meaabaqaciaacaGaaeqabaqabeGadaaakeaacqWGdbWqdaqhaaWcbaacciGae8xSdegabaGafm4AaSMbaSaacqGGSaalcqWGPbqAaaaaaa@333A@ denotes the 3-D coordinate of the *i*^*th *^*C*_*α *_atom. Protein backbones can be transformed into 2-D distance matrices. For Ω^*k*^, the corresponding distance matrix, *D*^*k*^, is defined as *D*[*i*, *j*] = *dist*(Cαk→,i
 MathType@MTEF@5@5@+=feaafiart1ev1aaatCvAUfKttLearuWrP9MDH5MBPbIqV92AaeXatLxBI9gBaebbnrfifHhDYfgasaacH8akY=wiFfYdH8Gipec8Eeeu0xXdbba9frFj0=OqFfea0dXdd9vqai=hGuQ8kuc9pgc9s8qqaq=dirpe0xb9q8qiLsFr0=vr0=vr0dc8meaabaqaciaacaGaaeqabaqabeGadaaakeaacqWGdbWqdaqhaaWcbaacciGae8xSdegabaGafm4AaSMbaSaacqGGSaalcqWGPbqAaaaaaa@333A@, Cαk→,j
 MathType@MTEF@5@5@+=feaafiart1ev1aaatCvAUfKttLearuWrP9MDH5MBPbIqV92AaeXatLxBI9gBaebbnrfifHhDYfgasaacH8akY=wiFfYdH8Gipec8Eeeu0xXdbba9frFj0=OqFfea0dXdd9vqai=hGuQ8kuc9pgc9s8qqaq=dirpe0xb9q8qiLsFr0=vr0=vr0dc8meaabaqaciaacaGaaeqabaqabeGadaaakeaacqWGdbWqdaqhaaWcbaacciGae8xSdegabaGafm4AaSMbaSaacqGGSaalcqWGQbGAaaaaaa@333C@), 1 ≤ *i*, *j *≤ *n*, in a Euclidean space. A distance geometry method [29] shows that the 2-D distance matrix is generally sufficient to recover the original 3-D structure in polynomial time. Several examples in the literature convert protein backbone structures into distance matrices and then detect the structural similarity from them [9,30,31]. Since 2-D distance matrices maintain sufficient 3-D structural information, similar protein backbones are expected to have similar distance matrices. Figure 8 shows 3-D protein backbone structures and their corresponding 2-D distance matrices sampled from the SCOP *Heme-dependent peroxidases *and *Acid proteases *folds. Within each SCOP fold, the proteins maintain high similarities in both 3-D backbone structures and 2-D distance matrices. Variations in distance matrices are detectable by comparing structures that belong to different folds.

#### Feature extraction

In the area of content-based image retrieval (*CBIR*), several computational techniques have been developed to retrieve visually similar images from databases for a query image [32-34]. When each element of the distance matrix is interpreted as a grayscale pixel, the distance matrix can be converted into a 2-D image. After preprocessing protein structures into grayscale images, we apply the *CBIR *technique to retrieve similar distance matrices in ranked order. In our previous work [17,19], we extract 24 local and 9 global features that are relevant to the visual content of distance matrices using a suite of computer vision algorithms such as histograms and textures [35-37]. To obtain local features, each distance matrix is partitioned into six band regions, which are parallel to the diagonal of the matrix. In each band, histograms are computed by four bins of distance ranges: [0–5], [6–10], [11–15], and [16-∞]. Since the distance matrix is symmetric, global features such as *Entropy, Homogenity *and *Contrast *are computed for the entire upper triangle of each distance image. Interested readers are referred to our previous publications [17,19] for details of the feature extraction algorithms applied in this work. The transformation of a distance matrix into a 33-D feature vector ensures each feature vector uniquely identifies a protein chain. Table 5 and Table 6 list the 24 local features and 9 global features extracted for proteins from the SCOP *Heme-dependent peroxidases *and *Acid proteases *folds, respectively. For a large-scale protein database such as the SCOP database, it is important to develop a classifier that groups proteins within the same fold and separates proteins from different folds.

#### Fold assignment

To ensure high accuracy when classifying a newly-discovered protein, we have designed a novel method that extends algorithms from Information Retrieval (*IR*) [38]. For the assignment of newly-discovered proteins to the known folds, we first discuss two well-recognized methods, *C4.5 Decision Tree (DT) *[26] and *Nearest Neighbor *(NN) [25], and then our new approach, *E-Predict*, which achieves a better classification accuracy than *C4.5 DT *or NN.

Decision tree approaches have been developed for classification in supervised machine learning [26]. Using a set of ground truth data that contains feature vectors of proteins and their associated fold labels, a classifier usually divides the high-dimensional feature space, discussed previously, into multiple subspaces, which are normally in the form of *hyper-cubes *or *hyper-spheres*. In the labeling process using *C4.5 DT*, the majority of proteins from the same fold are expected to be clustered into a small number of subspaces. Proteins from different folds are separated into different subspaces based on minimization of entropy. A newly-discovered protein can then be classified into one of the known subspaces for fold assignment by following decision features of internal tree nodes and their corresponding thresholds. However, a small number of proteins from the same SCOP fold with similar feature values may be partitioned into different leaf nodes by *C4.5 DT *due to their feature values, which are distributed around thresholds of internal nodes. With hundreds of folds in the SCOP database, the more proteins from different folds that have been grouped into a leaf node, the higher the probability of misclassification.

**Figure 9:**
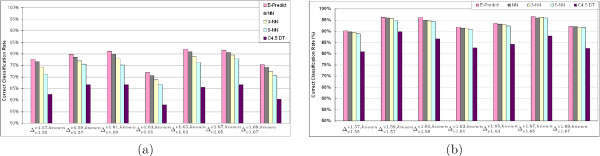
A comparison of classification performance between *E-Predict, NN*, 3-NN, 5-NN, and *C4.5 DT *classifiers using (a) testing proteins in Δv1v2,known
 MathType@MTEF@5@5@+=feaafiart1ev1aaatCvAUfKttLearuWrP9MDH5MBPbIqV92AaeXatLxBI9gBaebbnrfifHhDYfgasaacH8akY=wiFfYdH8Gipec8Eeeu0xXdbba9frFj0=OqFfea0dXdd9vqai=hGuQ8kuc9pgc9s8qqaq=dirpe0xb9q8qiLsFr0=vr0=vr0dc8meaabaqaciaacaGaaeqabaqabeGadaaakeaacqqHuoardaqhaaWcbaGaemODay3aaSbaaWqaaiabigdaXaqabaaaleaacqWG2bGDdaWgaaadbaGaeGOmaidabeaaliabcYcaSiabdUgaRjabd6gaUjabd+gaVjabdEha3jabd6gaUbaaaaa@3B62@ which are selected from the SCOP folds in *v*_2 _that have at least one protein in *v*_1 _(b) testing proteins in Δv1v2,known
 MathType@MTEF@5@5@+=feaafiart1ev1aaatCvAUfKttLearuWrP9MDH5MBPbIqV92AaeXatLxBI9gBaebbnrfifHhDYfgasaacH8akY=wiFfYdH8Gipec8Eeeu0xXdbba9frFj0=OqFfea0dXdd9vqai=hGuQ8kuc9pgc9s8qqaq=dirpe0xb9q8qiLsFr0=vr0=vr0dc8meaabaqaciaacaGaaeqabaqabeGadaaakeaacqqHuoardaqhaaWcbaGaemODay3aaSbaaWqaaiabigdaXaqabaaaleaacqWG2bGDdaWgaaadbaGaeGOmaidabeaaliabcYcaSiabdUgaRjabd6gaUjabd+gaVjabdEha3jabd6gaUbaaaaa@3B62@ which are selected from the SCOP folds in *v*_2 _that have at least 10 proteins in *v*_1_.

**Figure 10:**
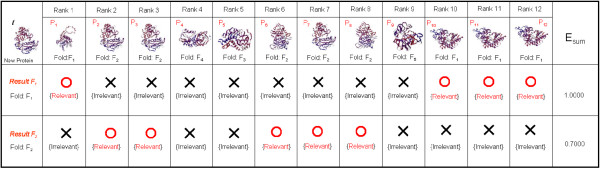
An example of *E_Measure *calculations for two SCOP folds in a list of nearest neighbor proteins.

Instead of partitioning the high-dimensional space, *Nearest Neighbor *(NN) [25] assigns a SCOP fold for a newly-discovered protein by searching for its nearest neighbor with the Euclidean distance measurement. Figure 9(a) shows that NN outperforms *C4.5 DT *by 13%, on average, for fold assignments using the test sets in Δv1v2,known
 MathType@MTEF@5@5@+=feaafiart1ev1aaatCvAUfKttLearuWrP9MDH5MBPbIqV92AaeXatLxBI9gBaebbnrfifHhDYfgasaacH8akY=wiFfYdH8Gipec8Eeeu0xXdbba9frFj0=OqFfea0dXdd9vqai=hGuQ8kuc9pgc9s8qqaq=dirpe0xb9q8qiLsFr0=vr0=vr0dc8meaabaqaciaacaGaaeqabaqabeGadaaakeaacqqHuoardaqhaaWcbaGaemODay3aaSbaaWqaaiabigdaXaqabaaaleaacqWG2bGDdaWgaaadbaGaeGOmaidabeaaliabcYcaSiabdUgaRjabd6gaUjabd+gaVjabdEha3jabd6gaUbaaaaa@3B62@, which are selected from the SCOP folds in the SCOP *v*_2 _release that have at least one protein from the SCOP *v*_1 _release. Figure 9(b) shows that NN also outperforms *C4.5 DT *by 8.45%, on average, for fold assignments using the test sets in Δv1v2,known
 MathType@MTEF@5@5@+=feaafiart1ev1aaatCvAUfKttLearuWrP9MDH5MBPbIqV92AaeXatLxBI9gBaebbnrfifHhDYfgasaacH8akY=wiFfYdH8Gipec8Eeeu0xXdbba9frFj0=OqFfea0dXdd9vqai=hGuQ8kuc9pgc9s8qqaq=dirpe0xb9q8qiLsFr0=vr0=vr0dc8meaabaqaciaacaGaaeqabaqabeGadaaakeaacqqHuoardaqhaaWcbaGaemODay3aaSbaaWqaaiabigdaXaqabaaaleaacqWG2bGDdaWgaaadbaGaeGOmaidabeaaliabcYcaSiabdUgaRjabd6gaUjabd+gaVjabdEha3jabd6gaUbaaaaa@3B62@, which are selected from the SCOP folds in the SCOP *v*_2 _release that have at least 10 proteins from the SCOP *v*_1 _release. Even though NN yields a better classification performance than *C4.5 DT*, there still exists an important issue to consider: misclassifications from an outlier in NN search. An outlier is defined as a protein chain whose feature vector deviates greatly from the majority of proteins in the same SCOP fold. In the high-dimensional feature space with multiple overlapping SCOP folds, NN search may assign an incorrect SCOP fold to a newly-discovered protein by selecting an outlier as the nearest neighbor. For instance, we assume that the true fold of a newly-discovered protein *t *is *F*_2_. From *Result F*_1_, shown in the second row of Figure 10, the nearest neighbor of t is *p*_1_, which is an outlier to the majority of proteins in fold *F*_1_. When NN search is used for classification, the algorithm falsely classifies that *t *is in fold *F*_1_. One possible way to address this issue is to assign the newly-discovered protein to the SCOP fold that has the majority in the top *k Nearest Neighbor *(*k*-NN). In Figure 9(b), 3-NN yields a better accuracy than 5-NN in six test sets. Also, we find that 3-NN achieves a better accuracy than NN in Δv1.65v1.67,known
 MathType@MTEF@5@5@+=feaafiart1ev1aaatCvAUfKttLearuWrP9MDH5MBPbIqV92AaeXatLxBI9gBaebbnrfifHhDYfgasaacH8akY=wiFfYdH8Gipec8Eeeu0xXdbba9frFj0=OqFfea0dXdd9vqai=hGuQ8kuc9pgc9s8qqaq=dirpe0xb9q8qiLsFr0=vr0=vr0dc8meaabaqaciaacaGaaeqabaqabeGadaaakeaacqqHuoardaqhaaWcbaGaemODayNaeGymaeJaeiOla4IaeGOnayJaeGynaudabaGaemODayNaeGymaeJaeiOla4IaeGOnayJaeG4naCJaeiilaWIaem4AaSMaemOBa4Maem4Ba8Maem4DaCNaemOBa4gaaaaa@40A0@. Unfortunately, 3-NN does not perform as well as NN on the other test sets due to the existence of two or more outliers in the 3-NN selection. In general, the *k*-NN classification method simply takes the majority of the top *k *nearest neighbors without considering the ranking information of nearest neighbor proteins.

In this work, we have developed the *E-Predict *algorithm which applies the *E*_*Measure *metric [21] to calculate the ranking information of nearest neighbor proteins. *E_Measure *was originally developed to evaluate the effectiveness of retrieval systems in *IR*. The more *relevant *documents retrieved with high ranks, the higher the retrieval accuracy. In the context of *IR, Precision *and *Recall *are two commonly used metrics for evaluating the retrieval performance. Let *n*_*t *_be the total number of *relevant *documents in the database for a certain query *t *and *s*(*R*,*i*) be the rank of the top *i*^*th *^*relevant *document in the retrieved document set *R *with 1 ≤ *i *≤ *n*_*t*_. *Precision *can be obtained by computing the ratio of the number of *relevant *documents retrieved to the total number of documents retrieved.

Precision(i)=is(R,i)     (3)
 MathType@MTEF@5@5@+=feaafiart1ev1aaatCvAUfKttLearuWrP9MDH5MBPbIqV92AaeXatLxBI9gBaebbnrfifHhDYfgasaacH8akY=wiFfYdH8Gipec8Eeeu0xXdbba9frFj0=OqFfea0dXdd9vqai=hGuQ8kuc9pgc9s8qqaq=dirpe0xb9q8qiLsFr0=vr0=vr0dc8meaabaqaciaacaGaaeqabaqabeGadaaakeaaieGacqWFqbaucqWFYbGCcqWGLbqzcqWGJbWycqWGPbqAcqWGZbWCcqWGPbqAcqWGVbWBcqWGUbGBcqGGOaakcqWGPbqAcqGGPaqkcqGH9aqpdaWcaaqaaiabdMgaPbqaaiabdohaZjabcIcaOiabdkfasjabcYcaSiabdMgaPjabcMcaPaaacaWLjaGaaCzcamaabmGabaGaeG4mamdacaGLOaGaayzkaaaaaa@48A2@

For example, if the second *relevant *document is ranked seventh in *R*, (i.e. *s*(*R*, 2) = 7), then *Precision*(2) = 27
 MathType@MTEF@5@5@+=feaafiart1ev1aaatCvAUfKttLearuWrP9MDH5MBPbIqV92AaeXatLxBI9gBaebbnrfifHhDYfgasaacH8akY=wiFfYdH8Gipec8Eeeu0xXdbba9frFj0=OqFfea0dXdd9vqai=hGuQ8kuc9pgc9s8qqaq=dirpe0xb9q8qiLsFr0=vr0=vr0dc8meaabaqaciaacaGaaeqabaqabeGadaaakeaadaWcaaqaaiabikdaYaqaaiabiEda3aaaaaa@2EAA@. *Recall *is the ratio of the number of *relevant *documents *i *retrieved to the total number of *relevant *documents *n*_*t *_in the database.

Recall(i)=int     (4)
 MathType@MTEF@5@5@+=feaafiart1ev1aaatCvAUfKttLearuWrP9MDH5MBPbIqV92AaeXatLxBI9gBaebbnrfifHhDYfgasaacH8akY=wiFfYdH8Gipec8Eeeu0xXdbba9frFj0=OqFfea0dXdd9vqai=hGuQ8kuc9pgc9s8qqaq=dirpe0xb9q8qiLsFr0=vr0=vr0dc8meaabaqaciaacaGaaeqabaqabeGadaaakeaaieGacqWFsbGucqWFLbqzcqWGJbWycqWGHbqycqWGSbaBcqWGSbaBcqGGOaakcqWGPbqAcqGGPaqkcqGH9aqpdaWcaaqaaiabdMgaPbqaaiabd6gaUnaaBaaaleaacqWG0baDaeqaaaaakiaaxMaacaWLjaWaaeWaceaacqaI0aanaiaawIcacaGLPaaaaaa@40DA@

For example, if there exist 11 *relevant *documents for query *t*, the second *relevant *document in the results will have *Recall*(2) = 211
 MathType@MTEF@5@5@+=feaafiart1ev1aaatCvAUfKttLearuWrP9MDH5MBPbIqV92AaeXatLxBI9gBaebbnrfifHhDYfgasaacH8akY=wiFfYdH8Gipec8Eeeu0xXdbba9frFj0=OqFfea0dXdd9vqai=hGuQ8kuc9pgc9s8qqaq=dirpe0xb9q8qiLsFr0=vr0=vr0dc8meaabaqaciaacaGaaeqabaqabeGadaaakeaadaWcaaqaaiabikdaYaqaaiabigdaXiabigdaXaaaaaa@2F8E@. *E*_*Measure *takes into consideration both *Precision *and *Recall to *evaluate the retrieval accuracy with a weighting factor *b *as shown in the following equation:

E_Measure(i,b)=1−1+b21Precision(i)+b2Recall(i)     (5)
 MathType@MTEF@5@5@+=feaafiart1ev1aaatCvAUfKttLearuWrP9MDH5MBPbIqV92AaeXatLxBI9gBaebbnrfifHhDYfgasaacH8akY=wiFfYdH8Gipec8Eeeu0xXdbba9frFj0=OqFfea0dXdd9vqai=hGuQ8kuc9pgc9s8qqaq=dirpe0xb9q8qiLsFr0=vr0=vr0dc8meaabaqaciaacaGaaeqabaqabeGadaaakeaacqWGfbqrcqGGFbWxcqWGnbqtcqWGLbqzcqWGHbqycqWGZbWCcqWG1bqDcqWGYbGCcqWGLbqzcqGGOaakcqWGPbqAcqGGSaalcqWGIbGycqGGPaqkcqGH9aqpcqaIXaqmcqGHsisldaWcaaqaaiabigdaXiabgUcaRiabdkgaInaaCaaaleqabaGaeGOmaidaaaGcbaWaaSaaaeaacqaIXaqmaeaaieGacqWFqbaucqWFYbGCcqWGLbqzcqWGJbWycqWGPbqAcqWGZbWCcqWGPbqAcqWGVbWBcqWGUbGBcqGGOaakcqWGPbqAcqGGPaqkaaGaey4kaSYaaSaaaeaacqWGIbGydaahaaWcbeqaaiabikdaYaaaaOqaaiab=jfasjab=vgaLjabdogaJjabdggaHjabdYgaSjabdYgaSjabcIcaOiabdMgaPjabcMcaPaaaaaGaaCzcaiaaxMaadaqadiqaaiabiwda1aGaayjkaiaawMcaaaaa@6726@

When a *relevant *document is highly ranked, a low *E*_*Measure *is expected. The *effectiveness *of a retrieval system *ς *can be evaluated by the summation of *E_Measures *for all *n*_*t *_*relevant *documents.

Esumt(ς)=∑i=1ntE_Measure(i,b)     (6)
 MathType@MTEF@5@5@+=feaafiart1ev1aaatCvAUfKttLearuWrP9MDH5MBPbIqV92AaeXatLxBI9gBaebbnrfifHhDYfgasaacH8akY=wiFfYdH8Gipec8Eeeu0xXdbba9frFj0=OqFfea0dXdd9vqai=hGuQ8kuc9pgc9s8qqaq=dirpe0xb9q8qiLsFr0=vr0=vr0dc8meaabaqaciaacaGaaeqabaqabeGadaaakeaacqWGfbqrdaqhaaWcbaGaem4CamNaemyDauNaemyBa0gabaGaemiDaqhaaOGaeiikaGccciGae8NWdyLaeiykaKIaeyypa0ZaaabCaeaacqWGfbqrcqGGFbWxcqWGnbqtcqWGLbqzcqWGHbqycqWGZbWCcqWG1bqDcqWGYbGCcqWGLbqzaSqaaiabdMgaPjabg2da9iabigdaXaqaaiabd6gaUnaaBaaameaacqWG0baDaeqaaaqdcqGHris5aOGaeiikaGIaemyAaKMaeiilaWIaemOyaiMaeiykaKIaaCzcaiaaxMaadaqadiqaaiabiAda2aGaayjkaiaawMcaaaaa@556F@

In practice, the best *IR *system is the one with the smallest *E_sum*(*ς*).

Instead of directly applying the above-mentioned evaluation method for our SCOP fold classification task, our *E-Predict *algorithm extends the method by visiting candidate folds in the top *k *nearest neighbor results *R*, and then ranking the folds using *E*_*Measure*. The *E-Predict *algorithm is shown in Appendix 1. From lines 2 to 16, the algorithm collects the SCOP folds of retrieved proteins in *R *into a set of candidate SCOP folds, II, with each candidate fold having at least *n*_*t *_proteins appearing in *R*. The algorithm then computes an evaluation score Esumt
 MathType@MTEF@5@5@+=feaafiart1ev1aaatCvAUfKttLearuWrP9MDH5MBPbIqV92AaeXatLxBI9gBaebbnrfifHhDYfgasaacH8akY=wiFfYdH8Gipec8Eeeu0xXdbba9frFj0=OqFfea0dXdd9vqai=hGuQ8kuc9pgc9s8qqaq=dirpe0xb9q8qiLsFr0=vr0=vr0dc8meaabaqaciaacaGaaeqabaqabeGadaaakeaacqWGfbqrdaqhaaWcbaGaem4CamNaemyDauNaemyBa0gabaGaemiDaqhaaaaa@33A2@(*F*) for each candidate SCOP fold, *F *∈ ∏, by accumulating *E*_*Measures *of the top *n*_*t *_proteins labeled with *F*, as shown from lines 17 to 26. Our approach assumes that the most relevant SCOP fold assigned to a newly-discovered protein *t *should have proteins that are highly ranked in *R*. For example, if *F*_1 _∈ ∏ is the candidate SCOP fold to be evaluated, we revisit *R *by assigning the label '*relevant' *to proteins that are from *F*_1 _and the label '*irrelevant' *to those from folds other than *F*_1_. Among these *relevant *proteins, we select the top *n*_*t *_proteins and form *R*_*F*1 _for our classification process. The *Result F*_1 _in Figure 10 shows that the top two proteins (*n*_*t *_= 2) labeled with *F*_1 _are ranked at 1 and 10.

For fold *F*_1_, the pairs of (precision, recall) for these two proteins are (*Precision*(1) = 11
 MathType@MTEF@5@5@+=feaafiart1ev1aaatCvAUfKttLearuWrP9MDH5MBPbIqV92AaeXatLxBI9gBaebbnrfifHhDYfgasaacH8akY=wiFfYdH8Gipec8Eeeu0xXdbba9frFj0=OqFfea0dXdd9vqai=hGuQ8kuc9pgc9s8qqaq=dirpe0xb9q8qiLsFr0=vr0=vr0dc8meaabaqaciaacaGaaeqabaqabeGadaaakeaadaWcaaqaaiabigdaXaqaaiabigdaXaaaaaa@2E9C@, *Recall*(1) = 12
 MathType@MTEF@5@5@+=feaafiart1ev1aaatCvAUfKttLearuWrP9MDH5MBPbIqV92AaeXatLxBI9gBaebbnrfifHhDYfgasaacH8akY=wiFfYdH8Gipec8Eeeu0xXdbba9frFj0=OqFfea0dXdd9vqai=hGuQ8kuc9pgc9s8qqaq=dirpe0xb9q8qiLsFr0=vr0=vr0dc8meaabaqaciaacaGaaeqabaqabeGadaaakeaadaWcaaqaaiabigdaXaqaaiabikdaYaaaaaa@2E9E@) and (*Precision*(2) = 210
 MathType@MTEF@5@5@+=feaafiart1ev1aaatCvAUfKttLearuWrP9MDH5MBPbIqV92AaeXatLxBI9gBaebbnrfifHhDYfgasaacH8akY=wiFfYdH8Gipec8Eeeu0xXdbba9frFj0=OqFfea0dXdd9vqai=hGuQ8kuc9pgc9s8qqaq=dirpe0xb9q8qiLsFr0=vr0=vr0dc8meaabaqaciaacaGaaeqabaqabeGadaaakeaadaWcaaqaaiabikdaYaqaaiabigdaXiabicdaWaaaaaa@2F8C@, *Recall*(2) = 22
 MathType@MTEF@5@5@+=feaafiart1ev1aaatCvAUfKttLearuWrP9MDH5MBPbIqV92AaeXatLxBI9gBaebbnrfifHhDYfgasaacH8akY=wiFfYdH8Gipec8Eeeu0xXdbba9frFj0=OqFfea0dXdd9vqai=hGuQ8kuc9pgc9s8qqaq=dirpe0xb9q8qiLsFr0=vr0=vr0dc8meaabaqaciaacaGaaeqabaqabeGadaaakeaadaWcaaqaaiabikdaYaqaaiabikdaYaaaaaa@2EA0@). Applying Eq.(5) with *b *= 1.0, we obtain *E_Measure*(1, 1.0) = 13
 MathType@MTEF@5@5@+=feaafiart1ev1aaatCvAUfKttLearuWrP9MDH5MBPbIqV92AaeXatLxBI9gBaebbnrfifHhDYfgasaacH8akY=wiFfYdH8Gipec8Eeeu0xXdbba9frFj0=OqFfea0dXdd9vqai=hGuQ8kuc9pgc9s8qqaq=dirpe0xb9q8qiLsFr0=vr0=vr0dc8meaabaqaciaacaGaaeqabaqabeGadaaakeaadaWcaaqaaiabigdaXaqaaiabiodaZaaaaaa@2EA0@ and *E*_*Measure*(2, 1.0) = 23
 MathType@MTEF@5@5@+=feaafiart1ev1aaatCvAUfKttLearuWrP9MDH5MBPbIqV92AaeXatLxBI9gBaebbnrfifHhDYfgasaacH8akY=wiFfYdH8Gipec8Eeeu0xXdbba9frFj0=OqFfea0dXdd9vqai=hGuQ8kuc9pgc9s8qqaq=dirpe0xb9q8qiLsFr0=vr0=vr0dc8meaabaqaciaacaGaaeqabaqabeGadaaakeaadaWcaaqaaiabikdaYaqaaiabiodaZaaaaaa@2EA2@. Substituting these two values into Eq.(6), we compute Esumt(ςF1)
 MathType@MTEF@5@5@+=feaafiart1ev1aaatCvAUfKttLearuWrP9MDH5MBPbIqV92AaeXatLxBI9gBaebbnrfifHhDYfgasaacH8akY=wiFfYdH8Gipec8Eeeu0xXdbba9frFj0=OqFfea0dXdd9vqai=hGuQ8kuc9pgc9s8qqaq=dirpe0xb9q8qiLsFr0=vr0=vr0dc8meaabaqaciaacaGaaeqabaqabeGadaaakeaacqWGfbqrdaqhaaWcbaGaem4CamNaemyDauNaemyBa0gabaGaemiDaqhaaOWaaeWaceaaiiGacqWFcpGvdaWgaaWcbaGaemOray0aaSbaaWqaaiabigdaXaqabaaaleqaaaGccaGLOaGaayzkaaaaaa@3956@ = 1.00. Similarly, for candidate fold *F*_2_, using *Result F*_2 _of Figure 10, the effectiveness of *F*_2 _is Esumt(ςF2)
 MathType@MTEF@5@5@+=feaafiart1ev1aaatCvAUfKttLearuWrP9MDH5MBPbIqV92AaeXatLxBI9gBaebbnrfifHhDYfgasaacH8akY=wiFfYdH8Gipec8Eeeu0xXdbba9frFj0=OqFfea0dXdd9vqai=hGuQ8kuc9pgc9s8qqaq=dirpe0xb9q8qiLsFr0=vr0=vr0dc8meaabaqaciaacaGaaeqabaqabeGadaaakeaacqWGfbqrdaqhaaWcbaGaem4CamNaemyDauNaemyBa0gabaGaemiDaqhaaOWaaeWaceaaiiGacqWFcpGvdaWgaaWcbaGaemOray0aaSbaaWqaaiabikdaYaqabaaaleqaaaGccaGLOaGaayzkaaaaaa@3958@ = 0.70.

According to Figure 3, there exists a significant number of small-size folds in the SCOP *v1*.69 release with 143 folds containing only one protein chain and 140 folds with two protein chains. When a newly-discovered protein belongs to a small-size fold, the algorithm might give a false positive due to insufficient ground truth data. To classify proteins in these small-size folds, we expect the NN search to retrieve a correct fold in the high-dimensional space by turning on a parameter λ in the *E-Predict *algorithm. Let *P*_0 _be the nearest neighbor protein of a query *t *in *R *and PNNF*
 MathType@MTEF@5@5@+=feaafiart1ev1aaatCvAUfKttLearuWrP9MDH5MBPbIqV92AaeXatLxBI9gBaebbnrfifHhDYfgasaacH8akY=wiFfYdH8Gipec8Eeeu0xXdbba9frFj0=OqFfea0dXdd9vqai=hGuQ8kuc9pgc9s8qqaq=dirpe0xb9q8qiLsFr0=vr0=vr0dc8meaabaqaciaacaGaaeqabaqabeGadaaakeaacqWGqbaudaqhaaWcbaGaemOta4KaemOta4eabaGaemOrayKaeiOkaOcaaaaa@323D@ be the nearest neighbor protein in the candidate fold with the minimum Esumt
 MathType@MTEF@5@5@+=feaafiart1ev1aaatCvAUfKttLearuWrP9MDH5MBPbIqV92AaeXatLxBI9gBaebbnrfifHhDYfgasaacH8akY=wiFfYdH8Gipec8Eeeu0xXdbba9frFj0=OqFfea0dXdd9vqai=hGuQ8kuc9pgc9s8qqaq=dirpe0xb9q8qiLsFr0=vr0=vr0dc8meaabaqaciaacaGaaeqabaqabeGadaaakeaacqWGfbqrdaqhaaWcbaGaem4CamNaemyDauNaemyBa0gabaGaemiDaqhaaaaa@33A2@ score (see line 28 of Appendix 1). The algorithm computes the structural variation values, *S*, for one pair (*t*,*P*_0_) and the other pair (*t*, PNNF*
 MathType@MTEF@5@5@+=feaafiart1ev1aaatCvAUfKttLearuWrP9MDH5MBPbIqV92AaeXatLxBI9gBaebbnrfifHhDYfgasaacH8akY=wiFfYdH8Gipec8Eeeu0xXdbba9frFj0=OqFfea0dXdd9vqai=hGuQ8kuc9pgc9s8qqaq=dirpe0xb9q8qiLsFr0=vr0=vr0dc8meaabaqaciaacaGaaeqabaqabeGadaaakeaacqWGqbaudaqhaaWcbaGaemOta4KaemOta4eabaGaemOrayKaeiOkaOcaaaaa@323D@) using the function in Eq.(2). The algorithm finally assigns the candidate fold with the minimum *S *value to the newly-discovered protein.

In the *E-Predict *algorithm, there exist two parameters, *b *and *n*_*t*_, that affect classification results. From our empirical observations, the best setting for the latest SCOP *v*1.69 release has *b *= 1.5 and *n*_*t *_= 6 with λ = *on *and *k *set to 500 nearest neighbors. Figure 9 shows comparisons of classification accuracies among *E-Predict*, NN, 3-NN, 5-NN, and *C4.5 DT *across seven test sets from Δv1.55v1.57,known
 MathType@MTEF@5@5@+=feaafiart1ev1aaatCvAUfKttLearuWrP9MDH5MBPbIqV92AaeXatLxBI9gBaebbnrfifHhDYfgasaacH8akY=wiFfYdH8Gipec8Eeeu0xXdbba9frFj0=OqFfea0dXdd9vqai=hGuQ8kuc9pgc9s8qqaq=dirpe0xb9q8qiLsFr0=vr0=vr0dc8meaabaqaciaacaGaaeqabaqabeGadaaakeaacqqHuoardaqhaaWcbaGaemODayNaeGymaeJaeiOla4IaeGynauJaeGynaudabaGaemODayNaeGymaeJaeiOla4IaeGynauJaeG4naCJaeiilaWIaem4AaSMaemOBa4Maem4Ba8Maem4DaCNaemOBa4gaaaaa@409C@ to Δv1.67v1.69,known
 MathType@MTEF@5@5@+=feaafiart1ev1aaatCvAUfKttLearuWrP9MDH5MBPbIqV92AaeXatLxBI9gBaebbnrfifHhDYfgasaacH8akY=wiFfYdH8Gipec8Eeeu0xXdbba9frFj0=OqFfea0dXdd9vqai=hGuQ8kuc9pgc9s8qqaq=dirpe0xb9q8qiLsFr0=vr0=vr0dc8meaabaqaciaacaGaaeqabaqabeGadaaakeaacqqHuoardaqhaaWcbaGaemODayNaeGymaeJaeiOla4IaeGOnayJaeG4naCdabaGaemODayNaeGymaeJaeiOla4IaeGOnayJaeGyoaKJaeiilaWIaem4AaSMaemOBa4Maem4Ba8Maem4DaCNaemOBa4gaaaaa@40A8@. For all test sets, *E-Predict *always outperforms *k*-NN and *C4.5 DT *with an improved classification accuracy.

### Recognizing the novel folds for newly-discovered proteins

**Figure 11:**
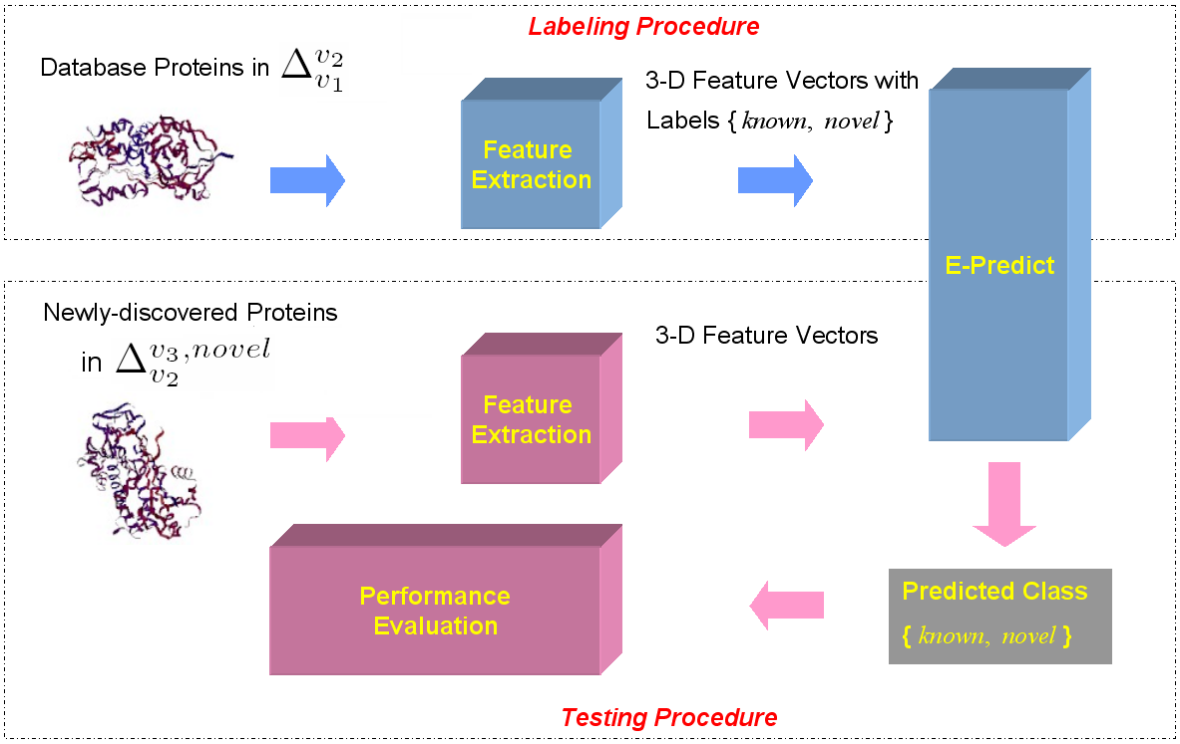
*E-Predict *model for recognizing the novel folds for newly-discovered proteins.

Classifying newly-discovered proteins into either the *novel folds *or the *known folds *has been identified as a two-class recognition problem [13]. Let *v*_1_, *v*_2 _and *v*_3 _denote three different SCOP releases in chronological order. To classify proteins from Δv2v3,novel
 MathType@MTEF@5@5@+=feaafiart1ev1aaatCvAUfKttLearuWrP9MDH5MBPbIqV92AaeXatLxBI9gBaebbnrfifHhDYfgasaacH8akY=wiFfYdH8Gipec8Eeeu0xXdbba9frFj0=OqFfea0dXdd9vqai=hGuQ8kuc9pgc9s8qqaq=dirpe0xb9q8qiLsFr0=vr0=vr0dc8meaabaqaciaacaGaaeqabaqabeGadaaakeaacqqHuoardaqhaaWcbaGaemODay3aaSbaaWqaaiabikdaYaqabaaaleaacqWG2bGDdaWgaaadbaGaeG4mamdabeaaliabcYcaSiabd6gaUjabd+gaVjabdAha2jabdwgaLjabdYgaSbaaaaa@3B54@, our algorithm relies on ground truth data from Δv1v2
 MathType@MTEF@5@5@+=feaafiart1ev1aaatCvAUfKttLearuWrP9MDH5MBPbIqV92AaeXatLxBI9gBaebbnrfifHhDYfgasaacH8akY=wiFfYdH8Gipec8Eeeu0xXdbba9frFj0=OqFfea0dXdd9vqai=hGuQ8kuc9pgc9s8qqaq=dirpe0xb9q8qiLsFr0=vr0=vr0dc8meaabaqaciaacaGaaeqabaqabeGadaaakeaacqqHuoardaqhaaWcbaGaemODay3aaSbaaWqaaiabigdaXaqabaaaleaacqWG2bGDdaWgaaadbaGaeGOmaidabeaaaaaaaa@3370@ with three features, which are derived from the result of *E-Predict *algorithm and will be discussed in great detail in the following section. In the labeling procedure of Figure 11, the algorithm first extracts the three features from proteins in Δv1v2
 MathType@MTEF@5@5@+=feaafiart1ev1aaatCvAUfKttLearuWrP9MDH5MBPbIqV92AaeXatLxBI9gBaebbnrfifHhDYfgasaacH8akY=wiFfYdH8Gipec8Eeeu0xXdbba9frFj0=OqFfea0dXdd9vqai=hGuQ8kuc9pgc9s8qqaq=dirpe0xb9q8qiLsFr0=vr0=vr0dc8meaabaqaciaacaGaaeqabaqabeGadaaakeaacqqHuoardaqhaaWcbaGaemODay3aaSbaaWqaaiabigdaXaqabaaaleaacqWG2bGDdaWgaaadbaGaeGOmaidabeaaaaaaaa@3370@. These proteins are then categorized into either the *known folds *of *v*_1 _or the *novel folds *as our ground truth data. In the testing procedure, proteins in the *novel folds *of Δv2v3,novel
 MathType@MTEF@5@5@+=feaafiart1ev1aaatCvAUfKttLearuWrP9MDH5MBPbIqV92AaeXatLxBI9gBaebbnrfifHhDYfgasaacH8akY=wiFfYdH8Gipec8Eeeu0xXdbba9frFj0=OqFfea0dXdd9vqai=hGuQ8kuc9pgc9s8qqaq=dirpe0xb9q8qiLsFr0=vr0=vr0dc8meaabaqaciaacaGaaeqabaqabeGadaaakeaacqqHuoardaqhaaWcbaGaemODay3aaSbaaWqaaiabikdaYaqabaaaleaacqWG2bGDdaWgaaadbaGaeG4mamdabeaaliabcYcaSiabd6gaUjabd+gaVjabdAha2jabdwgaLjabdYgaSbaaaaa@3B54@ are selected as our test data and are disjoint with our ground truth data. Once the three features are extracted from the testing proteins, we apply the *E-Predict *algorithm to classify test proteins into either the *novel folds *or the *known folds*.

#### Feature extraction

**Figure 12:**
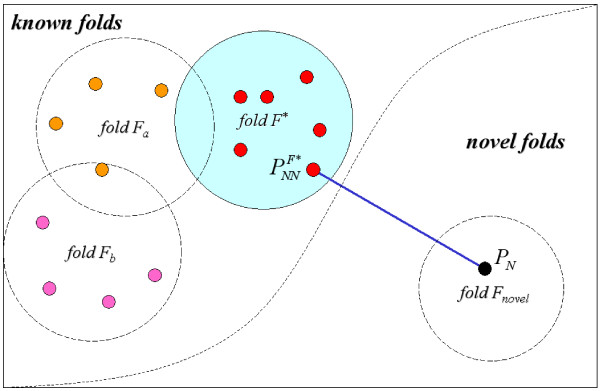
An example of identifying PNNF*
 MathType@MTEF@5@5@+=feaafiart1ev1aaatCvAUfKttLearuWrP9MDH5MBPbIqV92AaeXatLxBI9gBaebbnrfifHhDYfgasaacH8akY=wiFfYdH8Gipec8Eeeu0xXdbba9frFj0=OqFfea0dXdd9vqai=hGuQ8kuc9pgc9s8qqaq=dirpe0xb9q8qiLsFr0=vr0=vr0dc8meaabaqaciaacaGaaeqabaqabeGadaaakeaacqWGqbaudaqhaaWcbaGaemOta4KaemOta4eabaGaemOrayKaeiOkaOcaaaaa@323D@ for a newly-discovered protein *P*_*N *_in the *novel folds *by selecting the nearest neighbor protein in a fold F* derived from the *E-Predict *algorithm.

For a newly-discovered protein *P*_*N *_that does not belong to the *known folds*, we assume this protein has a low structural similarity to those proteins in the *known folds*. Under this assumption, we identify the three features that are used to achieve the novel fold recognition task. Figure 12 illustrates an example showing the three features for *P*_*N*_. The first feature, EsumPN(ςF*)
 MathType@MTEF@5@5@+=feaafiart1ev1aaatCvAUfKttLearuWrP9MDH5MBPbIqV92AaeXatLxBI9gBaebbnrfifHhDYfgasaacH8akY=wiFfYdH8Gipec8Eeeu0xXdbba9frFj0=OqFfea0dXdd9vqai=hGuQ8kuc9pgc9s8qqaq=dirpe0xb9q8qiLsFr0=vr0=vr0dc8meaabaqaciaacaGaaeqabaqabeGadaaakeaacqWGfbqrdaqhaaWcbaGaem4CamNaemyDauNaemyBa0gabaGaemiuaa1aaSbaaWqaaiabd6eaobqabaaaaOWaaeWaceaaiiGacqWFcpGvdaWgaaWcbaGaemOrayKaeiOkaOcabeaaaOGaayjkaiaawMcaaaaa@3A14@, is the minimum evaluation score of *P*_*N *_using the *E-Predict *algorithm with a suggested known fold F*. The second feature, *Dist*, represents the Euclidean distance between *P*_*N *_and PNNF*
 MathType@MTEF@5@5@+=feaafiart1ev1aaatCvAUfKttLearuWrP9MDH5MBPbIqV92AaeXatLxBI9gBaebbnrfifHhDYfgasaacH8akY=wiFfYdH8Gipec8Eeeu0xXdbba9frFj0=OqFfea0dXdd9vqai=hGuQ8kuc9pgc9s8qqaq=dirpe0xb9q8qiLsFr0=vr0=vr0dc8meaabaqaciaacaGaaeqabaqabeGadaaakeaacqWGqbaudaqhaaWcbaGaemOta4KaemOta4eabaGaemOrayKaeiOkaOcaaaaa@323D@, which denotes the nearest neighbor protein of *P*_*N *_labeled with fold F*. The third feature, *S*, is the structural variation value between *P*_*N *_and PNNF*
 MathType@MTEF@5@5@+=feaafiart1ev1aaatCvAUfKttLearuWrP9MDH5MBPbIqV92AaeXatLxBI9gBaebbnrfifHhDYfgasaacH8akY=wiFfYdH8Gipec8Eeeu0xXdbba9frFj0=OqFfea0dXdd9vqai=hGuQ8kuc9pgc9s8qqaq=dirpe0xb9q8qiLsFr0=vr0=vr0dc8meaabaqaciaacaGaaeqabaqabeGadaaakeaacqWGqbaudaqhaaWcbaGaemOta4KaemOta4eabaGaemOrayKaeiOkaOcaaaaa@323D@ using the function defined in Eq.(2). After feature extraction, these feature values are normalized between 0 and 1; each protein is then represented by a 3-D feature vector. The rationale for using these three features is in the following. Let *P*_*K *_be a newly-discovered protein that has been classified in the *known folds*. If *P*_*N *_is structurally dissimilar to all known protein structures from the SCOP database, then the Euclidean distance between *P*_*N *_and its nearest neighbor protein in a known fold suggested by *E-Predict *is expected to be greater than the distance between *P*_*K *_and its nearest neighbor protein PNNF*
 MathType@MTEF@5@5@+=feaafiart1ev1aaatCvAUfKttLearuWrP9MDH5MBPbIqV92AaeXatLxBI9gBaebbnrfifHhDYfgasaacH8akY=wiFfYdH8Gipec8Eeeu0xXdbba9frFj0=OqFfea0dXdd9vqai=hGuQ8kuc9pgc9s8qqaq=dirpe0xb9q8qiLsFr0=vr0=vr0dc8meaabaqaciaacaGaaeqabaqabeGadaaakeaacqWGqbaudaqhaaWcbaGaemOta4KaemOta4eabaGaemOrayKaeiOkaOcaaaaa@323D@. Similarly, the structural variation value of *P*_*N *_and its nearest neighbor protein is expected to be higher than the structural variation value of *P*_*K *_and its nearest neighbor protein. Also, the minimum evaluation score of *P*_*N*_, EsumPN(ςF*)
 MathType@MTEF@5@5@+=feaafiart1ev1aaatCvAUfKttLearuWrP9MDH5MBPbIqV92AaeXatLxBI9gBaebbnrfifHhDYfgasaacH8akY=wiFfYdH8Gipec8Eeeu0xXdbba9frFj0=OqFfea0dXdd9vqai=hGuQ8kuc9pgc9s8qqaq=dirpe0xb9q8qiLsFr0=vr0=vr0dc8meaabaqaciaacaGaaeqabaqabeGadaaakeaacqWGfbqrdaqhaaWcbaGaem4CamNaemyDauNaemyBa0gabaGaemiuaa1aaSbaaWqaaiabd6eaobqabaaaaOWaaeWaceaaiiGacqWFcpGvdaWgaaWcbaGaemOrayKaeiOkaOcabeaaaOGaayjkaiaawMcaaaaa@3A14@, is expected to be higher than the score of *P*_*K*_. Table 7 lists a brief summary of expected properties of the three features for proteins in the *novel folds *and the *known folds*.

#### Novel fold recognition

With the three features, labeling and testing procedures can be conducted to recognize the novel folds for newly-discovered proteins. According to the statistics in Table 2 for a certain release of the SCOP database, the majority of proteins are from the *known folds*. From our empirical observations, the classifier is biased to favor the *known folds *in a 3-D feature space with two overlapping classes. To reduce noise from the *known folds*, our model randomly selects an equal number of proteins from the *known folds *and the *novel folds *in the labeling procedure. We then apply the *E-Predict *algorithm to classify test proteins into either the *novel folds *or the *known folds*.

### Online index using the M-tree

Exhaustively searching for the nearest neighbors within a large-scale database such as the SCOP database is known to be computationally expensive. To improve the efficiency of *k*-NN search, we use an M-tree to index the high-dimensional feature vectors of proteins. The M-tree [27] scales well to support dynamic operations such as insert, delete and update. Each root node in a subtree maintains two values, a radius *R*_*s *_and a prototype protein, that create a *hyper-sphere *in the high-dimensional feature space, *A*_*s*_, to include all proteins within the subtree. During the Depth-First-Search(DFS) traversal, the M-tree algorithm maintains a priority queue with *k *slots to record the current *k *nearest neighbors. In addition, a radius centered at the query protein, *R*_*q*_, defines a search space *A*_*q*_. Initially, both *R*_*q *_and *A*_*q *_are set to ∞. Once a protein has been inserted into the queue, *R*_*q *_is then updated by the maximum Euclidean distance between the current proteins in the queue and the query protein, resulting in a much smaller search space *A*_*q*_. A fast nearest neighbor search is achieved by: 1) Applying the triangle inequality, the M-tree algorithm avoids traversing subtrees which do not overlap with the search space *A*_*q*_. 2) Concurrently, *A*_*q *_shrinks at a rapid rate due to the insertion of proteins in the queue. In our implementation, the M-tree indices have been properly organized into memory on several servers for a robust, fast nearest neighbor search.

### Web interface

**Figure 13:**
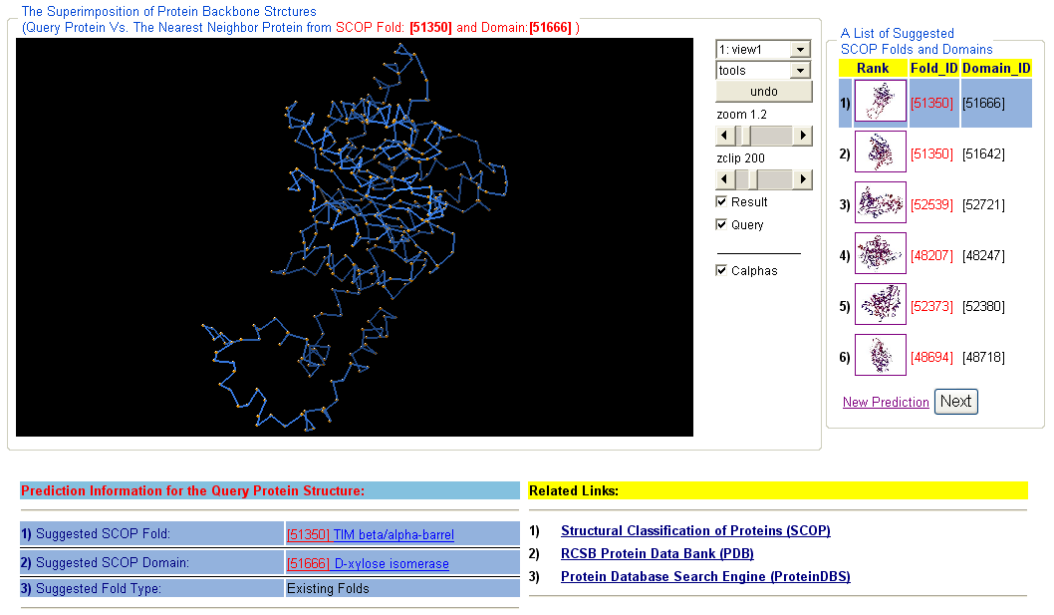
The superimposition of a newly-discovered protein and a known protein chain from the top ranked SCOP fold.

We have implemented a web interface to suggest a set of known SCOP folds and to recognize the novel folds for newly-discovered proteins. Users are allowed to submit 3-D protein structures in the PDB format. Our system first converts protein structures into 33-D feature vectors. Then, an evaluation score for each fold is computed from the ranked results of a nearest neighbors search. In seconds, a ranked list of SCOP folds is displayed to the user. To aid in visually inspecting the classification results, a tool is provided to superimpose a known protein from a suggested fold on the query structure using the *KiNG (Kinemage, Next Generation) *graphic package . In Figure 13, a 3-D superimposition view shows that the query protein is structurally similar to a known protein 9*xim_A *in the suggested fold. Our system, *ProteinDBS-predict*, is publicly accessible at .

## Authors' contributions

Both CS and PC designed the algorithm. PC implemented related programs and a web-based interface. CS supervised the whole project. DX contributed technical advice and helped test the system. PC drafted the manuscript and all authors finalized it. All authors read and approved the final manuscript.

## Appendix

## Figures and Tables

**Table 1:** A test set that contains 37 protein chains from Δv1.59v1.61,known
 MathType@MTEF@5@5@+=feaafiart1ev1aaatCvAUfKttLearuWrP9MDH5MBPbIqV92AaeXatLxBI9gBaebbnrfifHhDYfgasaacH8akY=wiFfYdH8Gipec8Eeeu0xXdbba9frFj0=OqFfea0dXdd9vqai=hGuQ8kuc9pgc9s8qqaq=dirpe0xb9q8qiLsFr0=vr0=vr0dc8meaabaqaciaacaGaaeqabaqabeGadaaakeaacqqHuoardaqhaaWcbaGaemODayNaeGymaeJaeiOla4IaeGynauJaeGyoaKdabaGaemODayNaeGymaeJaeiOla4IaeGOnayJaeGymaeJaeiilaWIaem4AaSMaemOBa4Maem4Ba8Maem4DaCNaemOBa4gaaaaa@409A@ [13].

*pdb*_*id*	*fold*_*id*	*pdb*_*id*	*fold*_*id*	*pdb*_*id*	*fold*_*id*	*pdb*_*id*	*fold*_*id*	*pdb*_*id*	*fold*_*id*
1*gyz*_*A*	63569	1*key*_*A*	48370	1*key*_*B*	48370	1*key*_*C*	48370	1*key*_*D*	48370
1*lkv*_*X*	48370	1*ldk*_*A*	48370	1*ifr*_*A*	48725	1*ivt*_*A*	48725	1*gyv*_*A*	48725
1*gyu*_*A*	48725	1*iu1*_*A*	48725	1*iu1*_*B*	48725	1*gyw*_*A*	48725	1*gyw*_*B*	48725
1*l6p*_*A*	48725	1*lpl*_*A*	50036	1*k*3*b*_*A*	50875	1*gyh*_*A*	50933	1*gyh*_*B*	50933
1*gyh*_*C*	50933	1*gyh*_*D*	50933	1*gyh*_*E*	50933	1*gyh*_*F*	50933	1*gyd*_*B*	50933
1*gye*_*B*	50933	1*jof*_*A*	50964	1*jof*_*B*	50964	1*jof*_*C*	50964	1*jof*_*D*	50964
1*jof*_*E*	50964	1*jof*_*F*	50964	1*jof*_*G*	50964	1*Jof*_*H*	50964	1*l*2*q*_*A*	51350
1*ln*4_*A*	55199	1*kuu*_*A*	56234						

**Table 2:** The number of proteins in a test set of novel folds, general and non-redundant test sets in Δv1v2,known
 MathType@MTEF@5@5@+=feaafiart1ev1aaatCvAUfKttLearuWrP9MDH5MBPbIqV92AaeXatLxBI9gBaebbnrfifHhDYfgasaacH8akY=wiFfYdH8Gipec8Eeeu0xXdbba9frFj0=OqFfea0dXdd9vqai=hGuQ8kuc9pgc9s8qqaq=dirpe0xb9q8qiLsFr0=vr0=vr0dc8meaabaqaciaacaGaaeqabaqabeGadaaakeaacqqHuoardaqhaaWcbaGaemODay3aaSbaaWqaaiabigdaXaqabaaaleaacqWG2bGDdaWgaaadbaGaeGOmaidabeaaliabcYcaSiabdUgaRjabd6gaUjabd+gaVjabdEha3jabd6gaUbaaaaa@3B62@ which are selected from the known SCOP folds of *v*_2 _with at least one protein chain in *v*_1_.

test set	size *(#proteins)*	test set	size *(#proteins)*	test set	size *(#proteins)*
Δv1.55,generalv1.57,known MathType@MTEF@5@5@+=feaafiart1ev1aaatCvAUfKttLearuWrP9MDH5MBPbIqV92AaeXatLxBI9gBaebbnrfifHhDYfgasaacH8akY=wiFfYdH8Gipec8Eeeu0xXdbba9frFj0=OqFfea0dXdd9vqai=hGuQ8kuc9pgc9s8qqaq=dirpe0xb9q8qiLsFr0=vr0=vr0dc8meaabaqaciaacaGaaeqabaqabeGadaaakeaacqqHuoardaqhaaWcbaGaemODayNaeGymaeJaeiOla4IaeGynauJaeGynauJaeiilaWIaem4zaCMaemyzauMaemOBa4MaemyzauMaemOCaiNaemyyaeMaemiBaWgabaGaemODayNaeGymaeJaeiOla4IaeGynauJaeG4naCJaeiilaWIaem4AaSMaemOBa4Maem4Ba8Maem4DaCNaemOBa4gaaaaa@4AF7@	4192	Δv1.55,non−redundantv1.57,known MathType@MTEF@5@5@+=feaafiart1ev1aaatCvAUfKttLearuWrP9MDH5MBPbIqV92AaeXatLxBI9gBaebbnrfifHhDYfgasaacH8akY=wiFfYdH8Gipec8Eeeu0xXdbba9frFj0=OqFfea0dXdd9vqai=hGuQ8kuc9pgc9s8qqaq=dirpe0xb9q8qiLsFr0=vr0=vr0dc8meaabaqaciaacaGaaeqabaqabeGadaaakeaacqqHuoardaqhaaWcbaGaemODayNaeGymaeJaeiOla4IaeGynauJaeGynauJaeiilaWIaemOBa4Maem4Ba8MaemOBa4MaeyOeI0IaemOCaiNaemyzauMaemizaqMaemyDauNaemOBa4MaemizaqMaemyyaeMaemOBa4MaemiDaqhabaGaemODayNaeGymaeJaeiOla4IaeGynauJaeG4naCJaeiilaWIaem4AaSMaemOBa4Maem4Ba8Maem4DaCNaemOBa4gaaaaa@52F5@	442	-	-
Δv1.57,generalv1.59,known MathType@MTEF@5@5@+=feaafiart1ev1aaatCvAUfKttLearuWrP9MDH5MBPbIqV92AaeXatLxBI9gBaebbnrfifHhDYfgasaacH8akY=wiFfYdH8Gipec8Eeeu0xXdbba9frFj0=OqFfea0dXdd9vqai=hGuQ8kuc9pgc9s8qqaq=dirpe0xb9q8qiLsFr0=vr0=vr0dc8meaabaqaciaacaGaaeqabaqabeGadaaakeaacqqHuoardaqhaaWcbaGaemODayNaeGymaeJaeiOla4IaeGynauJaeG4naCJaeiilaWIaem4zaCMaemyzauMaemOBa4MaemyzauMaemOCaiNaemyyaeMaemiBaWgabaGaemODayNaeGymaeJaeiOla4IaeGynauJaeGyoaKJaeiilaWIaem4AaSMaemOBa4Maem4Ba8Maem4DaCNaemOBa4gaaaaa@4AFF@	4047	Δv1.57,non−redundantv1.59,known MathType@MTEF@5@5@+=feaafiart1ev1aaatCvAUfKttLearuWrP9MDH5MBPbIqV92AaeXatLxBI9gBaebbnrfifHhDYfgasaacH8akY=wiFfYdH8Gipec8Eeeu0xXdbba9frFj0=OqFfea0dXdd9vqai=hGuQ8kuc9pgc9s8qqaq=dirpe0xb9q8qiLsFr0=vr0=vr0dc8meaabaqaciaacaGaaeqabaqabeGadaaakeaacqqHuoardaqhaaWcbaGaemODayNaeGymaeJaeiOla4IaeGynauJaeG4naCJaeiilaWIaemOBa4Maem4Ba8MaemOBa4MaeyOeI0IaemOCaiNaemyzauMaemizaqMaemyDauNaemOBa4MaemizaqMaemyyaeMaemOBa4MaemiDaqhabaGaemODayNaeGymaeJaeiOla4IaeGynauJaeGyoaKJaeiilaWIaem4AaSMaemOBa4Maem4Ba8Maem4DaCNaemOBa4gaaaaa@52FD@	431	Δv1.57v1.59,novel MathType@MTEF@5@5@+=feaafiart1ev1aaatCvAUfKttLearuWrP9MDH5MBPbIqV92AaeXatLxBI9gBaebbnrfifHhDYfgasaacH8akY=wiFfYdH8Gipec8Eeeu0xXdbba9frFj0=OqFfea0dXdd9vqai=hGuQ8kuc9pgc9s8qqaq=dirpe0xb9q8qiLsFr0=vr0=vr0dc8meaabaqaciaacaGaaeqabaqabeGadaaakeaacqqHuoardaqhaaWcbaGaemODayNaeGymaeJaeiOla4IaeGynauJaeG4naCdabaGaemODayNaeGymaeJaeiOla4IaeGynauJaeGyoaKJaeiilaWIaemOBa4Maem4Ba8MaemODayNaemyzauMaemiBaWgaaaaa@4092@	94
Δv1.59,generalv1.61,known MathType@MTEF@5@5@+=feaafiart1ev1aaatCvAUfKttLearuWrP9MDH5MBPbIqV92AaeXatLxBI9gBaebbnrfifHhDYfgasaacH8akY=wiFfYdH8Gipec8Eeeu0xXdbba9frFj0=OqFfea0dXdd9vqai=hGuQ8kuc9pgc9s8qqaq=dirpe0xb9q8qiLsFr0=vr0=vr0dc8meaabaqaciaacaGaaeqabaqabeGadaaakeaacqqHuoardaqhaaWcbaGaemODayNaeGymaeJaeiOla4IaeGynauJaeGyoaKJaeiilaWIaem4zaCMaemyzauMaemOBa4MaemyzauMaemOCaiNaemyyaeMaemiBaWgabaGaemODayNaeGymaeJaeiOla4IaeGOnayJaeGymaeJaeiilaWIaem4AaSMaemOBa4Maem4Ba8Maem4DaCNaemOBa4gaaaaa@4AF5@	4547	Δv1.59,non−redundantv1.61,known MathType@MTEF@5@5@+=feaafiart1ev1aaatCvAUfKttLearuWrP9MDH5MBPbIqV92AaeXatLxBI9gBaebbnrfifHhDYfgasaacH8akY=wiFfYdH8Gipec8Eeeu0xXdbba9frFj0=OqFfea0dXdd9vqai=hGuQ8kuc9pgc9s8qqaq=dirpe0xb9q8qiLsFr0=vr0=vr0dc8meaabaqaciaacaGaaeqabaqabeGadaaakeaacqqHuoardaqhaaWcbaGaemODayNaeGymaeJaeiOla4IaeGynauJaeGyoaKJaeiilaWIaemOBa4Maem4Ba8MaemOBa4MaeyOeI0IaemOCaiNaemyzauMaemizaqMaemyDauNaemOBa4MaemizaqMaemyyaeMaemOBa4MaemiDaqhabaGaemODayNaeGymaeJaeiOla4IaeGOnayJaeGymaeJaeiilaWIaem4AaSMaemOBa4Maem4Ba8Maem4DaCNaemOBa4gaaaaa@52F3@	468	Δv1.59v1.61,novel MathType@MTEF@5@5@+=feaafiart1ev1aaatCvAUfKttLearuWrP9MDH5MBPbIqV92AaeXatLxBI9gBaebbnrfifHhDYfgasaacH8akY=wiFfYdH8Gipec8Eeeu0xXdbba9frFj0=OqFfea0dXdd9vqai=hGuQ8kuc9pgc9s8qqaq=dirpe0xb9q8qiLsFr0=vr0=vr0dc8meaabaqaciaacaGaaeqabaqabeGadaaakeaacqqHuoardaqhaaWcbaGaemODayNaeGymaeJaeiOla4IaeGynauJaeGyoaKdabaGaemODayNaeGymaeJaeiOla4IaeGOnayJaeGymaeJaeiilaWIaemOBa4Maem4Ba8MaemODayNaemyzauMaemiBaWgaaaaa@4088@	10
Δv1.61,generalv1.63,known MathType@MTEF@5@5@+=feaafiart1ev1aaatCvAUfKttLearuWrP9MDH5MBPbIqV92AaeXatLxBI9gBaebbnrfifHhDYfgasaacH8akY=wiFfYdH8Gipec8Eeeu0xXdbba9frFj0=OqFfea0dXdd9vqai=hGuQ8kuc9pgc9s8qqaq=dirpe0xb9q8qiLsFr0=vr0=vr0dc8meaabaqaciaacaGaaeqabaqabeGadaaakeaacqqHuoardaqhaaWcbaGaemODayNaeGymaeJaeiOla4IaeGOnayJaeGymaeJaeiilaWIaem4zaCMaemyzauMaemOBa4MaemyzauMaemOCaiNaemyyaeMaemiBaWgabaGaemODayNaeGymaeJaeiOla4IaeGOnayJaeG4mamJaeiilaWIaem4AaSMaemOBa4Maem4Ba8Maem4DaCNaemOBa4gaaaaa@4AEB@	5226	Δv1.61,non−redundantv1.63known MathType@MTEF@5@5@+=feaafiart1ev1aaatCvAUfKttLearuWrP9MDH5MBPbIqV92AaeXatLxBI9gBaebbnrfifHhDYfgasaacH8akY=wiFfYdH8Gipec8Eeeu0xXdbba9frFj0=OqFfea0dXdd9vqai=hGuQ8kuc9pgc9s8qqaq=dirpe0xb9q8qiLsFr0=vr0=vr0dc8meaabaqaciaacaGaaeqabaqabeGadaaakeaacqqHuoardaqhaaWcbaGaemODayNaeGymaeJaeiOla4IaeGOnayJaeGymaeJaeiilaWIaemOBa4Maem4Ba8MaemOBa4MaeyOeI0IaemOCaiNaemyzauMaemizaqMaemyDauNaemOBa4MaemizaqMaemyyaeMaemOBa4MaemiDaqhabaGaemODayNaeGymaeJaeiOla4IaeGOnayJaeG4mamJaem4AaSMaemOBa4Maem4Ba8Maem4DaCNaemOBa4gaaaaa@5209@	491	Δv1.61v1.63,novel MathType@MTEF@5@5@+=feaafiart1ev1aaatCvAUfKttLearuWrP9MDH5MBPbIqV92AaeXatLxBI9gBaebbnrfifHhDYfgasaacH8akY=wiFfYdH8Gipec8Eeeu0xXdbba9frFj0=OqFfea0dXdd9vqai=hGuQ8kuc9pgc9s8qqaq=dirpe0xb9q8qiLsFr0=vr0=vr0dc8meaabaqaciaacaGaaeqabaqabeGadaaakeaacqqHuoardaqhaaWcbaGaemODayNaeGymaeJaeiOla4IaeGOnayJaeGymaedabaGaemODayNaeGymaeJaeiOla4IaeGOnayJaeG4mamJaeiilaWIaemOBa4Maem4Ba8MaemODayNaemyzauMaemiBaWgaaaaa@407E@	190
Δv1.63,generalv1.65,known MathType@MTEF@5@5@+=feaafiart1ev1aaatCvAUfKttLearuWrP9MDH5MBPbIqV92AaeXatLxBI9gBaebbnrfifHhDYfgasaacH8akY=wiFfYdH8Gipec8Eeeu0xXdbba9frFj0=OqFfea0dXdd9vqai=hGuQ8kuc9pgc9s8qqaq=dirpe0xb9q8qiLsFr0=vr0=vr0dc8meaabaqaciaacaGaaeqabaqabeGadaaakeaacqqHuoardaqhaaWcbaGaemODayNaeGymaeJaeiOla4IaeGOnayJaeG4mamJaeiilaWIaem4zaCMaemyzauMaemOBa4MaemyzauMaemOCaiNaemyyaeMaemiBaWgabaGaemODayNaeGymaeJaeiOla4IaeGOnayJaeGynauJaeiilaWIaem4AaSMaemOBa4Maem4Ba8Maem4DaCNaemOBa4gaaaaa@4AF3@	5445	Δv1.63,non−redundantv1.65,known MathType@MTEF@5@5@+=feaafiart1ev1aaatCvAUfKttLearuWrP9MDH5MBPbIqV92AaeXatLxBI9gBaebbnrfifHhDYfgasaacH8akY=wiFfYdH8Gipec8Eeeu0xXdbba9frFj0=OqFfea0dXdd9vqai=hGuQ8kuc9pgc9s8qqaq=dirpe0xb9q8qiLsFr0=vr0=vr0dc8meaabaqaciaacaGaaeqabaqabeGadaaakeaacqqHuoardaqhaaWcbaGaemODayNaeGymaeJaeiOla4IaeGOnayJaeG4mamJaeiilaWIaemOBa4Maem4Ba8MaemOBa4MaeyOeI0IaemOCaiNaemyzauMaemizaqMaemyDauNaemOBa4MaemizaqMaemyyaeMaemOBa4MaemiDaqhabaGaemODayNaeGymaeJaeiOla4IaeGOnayJaeGynauJaeiilaWIaem4AaSMaemOBa4Maem4Ba8Maem4DaCNaemOBa4gaaaaa@52F1@	494	Δv1.63v1.65,novel MathType@MTEF@5@5@+=feaafiart1ev1aaatCvAUfKttLearuWrP9MDH5MBPbIqV92AaeXatLxBI9gBaebbnrfifHhDYfgasaacH8akY=wiFfYdH8Gipec8Eeeu0xXdbba9frFj0=OqFfea0dXdd9vqai=hGuQ8kuc9pgc9s8qqaq=dirpe0xb9q8qiLsFr0=vr0=vr0dc8meaabaqaciaacaGaaeqabaqabeGadaaakeaacqqHuoardaqhaaWcbaGaemODayNaeGymaeJaeiOla4IaeGOnayJaeG4mamdabaGaemODayNaeGymaeJaeiOla4IaeGOnayJaeGynauJaeiilaWIaemOBa4Maem4Ba8MaemODayNaemyzauMaemiBaWgaaaaa@4086@	48
Δv1.65,generalv1.67,known MathType@MTEF@5@5@+=feaafiart1ev1aaatCvAUfKttLearuWrP9MDH5MBPbIqV92AaeXatLxBI9gBaebbnrfifHhDYfgasaacH8akY=wiFfYdH8Gipec8Eeeu0xXdbba9frFj0=OqFfea0dXdd9vqai=hGuQ8kuc9pgc9s8qqaq=dirpe0xb9q8qiLsFr0=vr0=vr0dc8meaabaqaciaacaGaaeqabaqabeGadaaakeaacqqHuoardaqhaaWcbaGaemODayNaeGymaeJaeiOla4IaeGOnayJaeGynauJaeiilaWIaem4zaCMaemyzauMaemOBa4MaemyzauMaemOCaiNaemyyaeMaemiBaWgabaGaemODayNaeGymaeJaeiOla4IaeGOnayJaeG4naCJaeiilaWIaem4AaSMaemOBa4Maem4Ba8Maem4DaCNaemOBa4gaaaaa@4AFB@	10521	Δv1.65,non−redundantv1.67,known MathType@MTEF@5@5@+=feaafiart1ev1aaatCvAUfKttLearuWrP9MDH5MBPbIqV92AaeXatLxBI9gBaebbnrfifHhDYfgasaacH8akY=wiFfYdH8Gipec8Eeeu0xXdbba9frFj0=OqFfea0dXdd9vqai=hGuQ8kuc9pgc9s8qqaq=dirpe0xb9q8qiLsFr0=vr0=vr0dc8meaabaqaciaacaGaaeqabaqabeGadaaakeaacqqHuoardaqhaaWcbaGaemODayNaeGymaeJaeiOla4IaeGOnayJaeGynauJaeiilaWIaemOBa4Maem4Ba8MaemOBa4MaeyOeI0IaemOCaiNaemyzauMaemizaqMaemyDauNaemOBa4MaemizaqMaemyyaeMaemOBa4MaemiDaqhabaGaemODayNaeGymaeJaeiOla4IaeGOnayJaeG4naCJaeiilaWIaem4AaSMaemOBa4Maem4Ba8Maem4DaCNaemOBa4gaaaaa@52F9@	736	Δv1.65v1.67,novel MathType@MTEF@5@5@+=feaafiart1ev1aaatCvAUfKttLearuWrP9MDH5MBPbIqV92AaeXatLxBI9gBaebbnrfifHhDYfgasaacH8akY=wiFfYdH8Gipec8Eeeu0xXdbba9frFj0=OqFfea0dXdd9vqai=hGuQ8kuc9pgc9s8qqaq=dirpe0xb9q8qiLsFr0=vr0=vr0dc8meaabaqaciaacaGaaeqabaqabeGadaaakeaacqqHuoardaqhaaWcbaGaemODayNaeGymaeJaeiOla4IaeGOnayJaeGynaudabaGaemODayNaeGymaeJaeiOla4IaeGOnayJaeG4naCJaeiilaWIaemOBa4Maem4Ba8MaemODayNaemyzauMaemiBaWgaaaaa@408E@	215
Δv1.67,generalv1.69,known MathType@MTEF@5@5@+=feaafiart1ev1aaatCvAUfKttLearuWrP9MDH5MBPbIqV92AaeXatLxBI9gBaebbnrfifHhDYfgasaacH8akY=wiFfYdH8Gipec8Eeeu0xXdbba9frFj0=OqFfea0dXdd9vqai=hGuQ8kuc9pgc9s8qqaq=dirpe0xb9q8qiLsFr0=vr0=vr0dc8meaabaqaciaacaGaaeqabaqabeGadaaakeaacqqHuoardaqhaaWcbaGaemODayNaeGymaeJaeiOla4IaeGOnayJaeG4naCJaeiilaWIaem4zaCMaemyzauMaemOBa4MaemyzauMaemOCaiNaemyyaeMaemiBaWgabaGaemODayNaeGymaeJaeiOla4IaeGOnayJaeGyoaKJaeiilaWIaem4AaSMaemOBa4Maem4Ba8Maem4DaCNaemOBa4gaaaaa@4B03@	5604	Δv1.67,non−redundantv1.69,known MathType@MTEF@5@5@+=feaafiart1ev1aaatCvAUfKttLearuWrP9MDH5MBPbIqV92AaeXatLxBI9gBaebbnrfifHhDYfgasaacH8akY=wiFfYdH8Gipec8Eeeu0xXdbba9frFj0=OqFfea0dXdd9vqai=hGuQ8kuc9pgc9s8qqaq=dirpe0xb9q8qiLsFr0=vr0=vr0dc8meaabaqaciaacaGaaeqabaqabeGadaaakeaacqqHuoardaqhaaWcbaGaemODayNaeGymaeJaeiOla4IaeGOnayJaeG4naCJaeiilaWIaemOBa4Maem4Ba8MaemOBa4MaeyOeI0IaemOCaiNaemyzauMaemizaqMaemyDauNaemOBa4MaemizaqMaemyyaeMaemOBa4MaemiDaqhabaGaemODayNaeGymaeJaeiOla4IaeGOnayJaeGyoaKJaeiilaWIaem4AaSMaemOBa4Maem4Ba8Maem4DaCNaemOBa4gaaaaa@5301@	585	Δv1.67v1.69,novel MathType@MTEF@5@5@+=feaafiart1ev1aaatCvAUfKttLearuWrP9MDH5MBPbIqV92AaeXatLxBI9gBaebbnrfifHhDYfgasaacH8akY=wiFfYdH8Gipec8Eeeu0xXdbba9frFj0=OqFfea0dXdd9vqai=hGuQ8kuc9pgc9s8qqaq=dirpe0xb9q8qiLsFr0=vr0=vr0dc8meaabaqaciaacaGaaeqabaqabeGadaaakeaacqqHuoardaqhaaWcbaGaemODayNaeGymaeJaeiOla4IaeGOnayJaeG4naCdabaGaemODayNaeGymaeJaeiOla4IaeGOnayJaeGyoaKJaeiilaWIaemOBa4Maem4Ba8MaemODayNaemyzauMaemiBaWgaaaaa@4096@	86

**Table 3:** The number of proteins in general and non-redundant test sets in Δv1v2,known
 MathType@MTEF@5@5@+=feaafiart1ev1aaatCvAUfKttLearuWrP9MDH5MBPbIqV92AaeXatLxBI9gBaebbnrfifHhDYfgasaacH8akY=wiFfYdH8Gipec8Eeeu0xXdbba9frFj0=OqFfea0dXdd9vqai=hGuQ8kuc9pgc9s8qqaq=dirpe0xb9q8qiLsFr0=vr0=vr0dc8meaabaqaciaacaGaaeqabaqabeGadaaakeaacqqHuoardaqhaaWcbaGaemODay3aaSbaaWqaaiabigdaXaqabaaaleaacqWG2bGDdaWgaaadbaGaeGOmaidabeaaliabcYcaSiabdUgaRjabd6gaUjabd+gaVjabdEha3jabd6gaUbaaaaa@3B62@ which are selected from the known SCOP folds of *v*_2 _with at least 10 protein chains in *v*_1_.

test set	size *(#proteins)*	test set	size *(#proteins)*
Δv1.55,generalv1.57,known MathType@MTEF@5@5@+=feaafiart1ev1aaatCvAUfKttLearuWrP9MDH5MBPbIqV92AaeXatLxBI9gBaebbnrfifHhDYfgasaacH8akY=wiFfYdH8Gipec8Eeeu0xXdbba9frFj0=OqFfea0dXdd9vqai=hGuQ8kuc9pgc9s8qqaq=dirpe0xb9q8qiLsFr0=vr0=vr0dc8meaabaqaciaacaGaaeqabaqabeGadaaakeaacqqHuoardaqhaaWcbaGaemODayNaeGymaeJaeiOla4IaeGynauJaeGynauJaeiilaWIaem4zaCMaemyzauMaemOBa4MaemyzauMaemOCaiNaemyyaeMaemiBaWgabaGaemODayNaeGymaeJaeiOla4IaeGynauJaeG4naCJaeiilaWIaem4AaSMaemOBa4Maem4Ba8Maem4DaCNaemOBa4gaaaaa@4AF7@	1832	Δv1.55,non−redundantv1.57,known MathType@MTEF@5@5@+=feaafiart1ev1aaatCvAUfKttLearuWrP9MDH5MBPbIqV92AaeXatLxBI9gBaebbnrfifHhDYfgasaacH8akY=wiFfYdH8Gipec8Eeeu0xXdbba9frFj0=OqFfea0dXdd9vqai=hGuQ8kuc9pgc9s8qqaq=dirpe0xb9q8qiLsFr0=vr0=vr0dc8meaabaqaciaacaGaaeqabaqabeGadaaakeaacqqHuoardaqhaaWcbaGaemODayNaeGymaeJaeiOla4IaeGynauJaeGynauJaeiilaWIaemOBa4Maem4Ba8MaemOBa4MaeyOeI0IaemOCaiNaemyzauMaemizaqMaemyDauNaemOBa4MaemizaqMaemyyaeMaemOBa4MaemiDaqhabaGaemODayNaeGymaeJaeiOla4IaeGynauJaeG4naCJaeiilaWIaem4AaSMaemOBa4Maem4Ba8Maem4DaCNaemOBa4gaaaaa@52F5@	158
Δv1.57,generalv1.59,known MathType@MTEF@5@5@+=feaafiart1ev1aaatCvAUfKttLearuWrP9MDH5MBPbIqV92AaeXatLxBI9gBaebbnrfifHhDYfgasaacH8akY=wiFfYdH8Gipec8Eeeu0xXdbba9frFj0=OqFfea0dXdd9vqai=hGuQ8kuc9pgc9s8qqaq=dirpe0xb9q8qiLsFr0=vr0=vr0dc8meaabaqaciaacaGaaeqabaqabeGadaaakeaacqqHuoardaqhaaWcbaGaemODayNaeGymaeJaeiOla4IaeGynauJaeG4naCJaeiilaWIaem4zaCMaemyzauMaemOBa4MaemyzauMaemOCaiNaemyyaeMaemiBaWgabaGaemODayNaeGymaeJaeiOla4IaeGynauJaeGyoaKJaeiilaWIaem4AaSMaemOBa4Maem4Ba8Maem4DaCNaemOBa4gaaaaa@4AFF@	1901	Δv1.57,non−redundantv1.59,known MathType@MTEF@5@5@+=feaafiart1ev1aaatCvAUfKttLearuWrP9MDH5MBPbIqV92AaeXatLxBI9gBaebbnrfifHhDYfgasaacH8akY=wiFfYdH8Gipec8Eeeu0xXdbba9frFj0=OqFfea0dXdd9vqai=hGuQ8kuc9pgc9s8qqaq=dirpe0xb9q8qiLsFr0=vr0=vr0dc8meaabaqaciaacaGaaeqabaqabeGadaaakeaacqqHuoardaqhaaWcbaGaemODayNaeGymaeJaeiOla4IaeGynauJaeG4naCJaeiilaWIaemOBa4Maem4Ba8MaemOBa4MaeyOeI0IaemOCaiNaemyzauMaemizaqMaemyDauNaemOBa4MaemizaqMaemyyaeMaemOBa4MaemiDaqhabaGaemODayNaeGymaeJaeiOla4IaeGynauJaeGyoaKJaeiilaWIaem4AaSMaemOBa4Maem4Ba8Maem4DaCNaemOBa4gaaaaa@52FD@	168
Δv1.59,generalv1.61,known MathType@MTEF@5@5@+=feaafiart1ev1aaatCvAUfKttLearuWrP9MDH5MBPbIqV92AaeXatLxBI9gBaebbnrfifHhDYfgasaacH8akY=wiFfYdH8Gipec8Eeeu0xXdbba9frFj0=OqFfea0dXdd9vqai=hGuQ8kuc9pgc9s8qqaq=dirpe0xb9q8qiLsFr0=vr0=vr0dc8meaabaqaciaacaGaaeqabaqabeGadaaakeaacqqHuoardaqhaaWcbaGaemODayNaeGymaeJaeiOla4IaeGynauJaeGyoaKJaeiilaWIaem4zaCMaemyzauMaemOBa4MaemyzauMaemOCaiNaemyyaeMaemiBaWgabaGaemODayNaeGymaeJaeiOla4IaeGOnayJaeGymaeJaeiilaWIaem4AaSMaemOBa4Maem4Ba8Maem4DaCNaemOBa4gaaaaa@4AF5@	2136	Δv1.59,non−redundantv1.61,known MathType@MTEF@5@5@+=feaafiart1ev1aaatCvAUfKttLearuWrP9MDH5MBPbIqV92AaeXatLxBI9gBaebbnrfifHhDYfgasaacH8akY=wiFfYdH8Gipec8Eeeu0xXdbba9frFj0=OqFfea0dXdd9vqai=hGuQ8kuc9pgc9s8qqaq=dirpe0xb9q8qiLsFr0=vr0=vr0dc8meaabaqaciaacaGaaeqabaqabeGadaaakeaacqqHuoardaqhaaWcbaGaemODayNaeGymaeJaeiOla4IaeGynauJaeGyoaKJaeiilaWIaemOBa4Maem4Ba8MaemOBa4MaeyOeI0IaemOCaiNaemyzauMaemizaqMaemyDauNaemOBa4MaemizaqMaemyyaeMaemOBa4MaemiDaqhabaGaemODayNaeGymaeJaeiOla4IaeGOnayJaeGymaeJaeiilaWIaem4AaSMaemOBa4Maem4Ba8Maem4DaCNaemOBa4gaaaaa@52F3@	166
Δv1.61,generalv1.63,known MathType@MTEF@5@5@+=feaafiart1ev1aaatCvAUfKttLearuWrP9MDH5MBPbIqV92AaeXatLxBI9gBaebbnrfifHhDYfgasaacH8akY=wiFfYdH8Gipec8Eeeu0xXdbba9frFj0=OqFfea0dXdd9vqai=hGuQ8kuc9pgc9s8qqaq=dirpe0xb9q8qiLsFr0=vr0=vr0dc8meaabaqaciaacaGaaeqabaqabeGadaaakeaacqqHuoardaqhaaWcbaGaemODayNaeGymaeJaeiOla4IaeGOnayJaeGymaeJaeiilaWIaem4zaCMaemyzauMaemOBa4MaemyzauMaemOCaiNaemyyaeMaemiBaWgabaGaemODayNaeGymaeJaeiOla4IaeGOnayJaeG4mamJaeiilaWIaem4AaSMaemOBa4Maem4Ba8Maem4DaCNaemOBa4gaaaaa@4AEB@	1947	Δv1.61,non−redundantv1.63known MathType@MTEF@5@5@+=feaafiart1ev1aaatCvAUfKttLearuWrP9MDH5MBPbIqV92AaeXatLxBI9gBaebbnrfifHhDYfgasaacH8akY=wiFfYdH8Gipec8Eeeu0xXdbba9frFj0=OqFfea0dXdd9vqai=hGuQ8kuc9pgc9s8qqaq=dirpe0xb9q8qiLsFr0=vr0=vr0dc8meaabaqaciaacaGaaeqabaqabeGadaaakeaacqqHuoardaqhaaWcbaGaemODayNaeGymaeJaeiOla4IaeGOnayJaeGymaeJaeiilaWIaemOBa4Maem4Ba8MaemOBa4MaeyOeI0IaemOCaiNaemyzauMaemizaqMaemyDauNaemOBa4MaemizaqMaemyyaeMaemOBa4MaemiDaqhabaGaemODayNaeGymaeJaeiOla4IaeGOnayJaeG4mamJaem4AaSMaemOBa4Maem4Ba8Maem4DaCNaemOBa4gaaaaa@5209@	189
Δv1.63,generalv1.65,known MathType@MTEF@5@5@+=feaafiart1ev1aaatCvAUfKttLearuWrP9MDH5MBPbIqV92AaeXatLxBI9gBaebbnrfifHhDYfgasaacH8akY=wiFfYdH8Gipec8Eeeu0xXdbba9frFj0=OqFfea0dXdd9vqai=hGuQ8kuc9pgc9s8qqaq=dirpe0xb9q8qiLsFr0=vr0=vr0dc8meaabaqaciaacaGaaeqabaqabeGadaaakeaacqqHuoardaqhaaWcbaGaemODayNaeGymaeJaeiOla4IaeGOnayJaeG4mamJaeiilaWIaem4zaCMaemyzauMaemOBa4MaemyzauMaemOCaiNaemyyaeMaemiBaWgabaGaemODayNaeGymaeJaeiOla4IaeGOnayJaeGynauJaeiilaWIaem4AaSMaemOBa4Maem4Ba8Maem4DaCNaemOBa4gaaaaa@4AF3@	2062	Δv1.63,non−redundantv1.65,known MathType@MTEF@5@5@+=feaafiart1ev1aaatCvAUfKttLearuWrP9MDH5MBPbIqV92AaeXatLxBI9gBaebbnrfifHhDYfgasaacH8akY=wiFfYdH8Gipec8Eeeu0xXdbba9frFj0=OqFfea0dXdd9vqai=hGuQ8kuc9pgc9s8qqaq=dirpe0xb9q8qiLsFr0=vr0=vr0dc8meaabaqaciaacaGaaeqabaqabeGadaaakeaacqqHuoardaqhaaWcbaGaemODayNaeGymaeJaeiOla4IaeGOnayJaeG4mamJaeiilaWIaemOBa4Maem4Ba8MaemOBa4MaeyOeI0IaemOCaiNaemyzauMaemizaqMaemyDauNaemOBa4MaemizaqMaemyyaeMaemOBa4MaemiDaqhabaGaemODayNaeGymaeJaeiOla4IaeGOnayJaeGynauJaeiilaWIaem4AaSMaemOBa4Maem4Ba8Maem4DaCNaemOBa4gaaaaa@52F1@	198
Δv1.65,generalv1.67,known MathType@MTEF@5@5@+=feaafiart1ev1aaatCvAUfKttLearuWrP9MDH5MBPbIqV92AaeXatLxBI9gBaebbnrfifHhDYfgasaacH8akY=wiFfYdH8Gipec8Eeeu0xXdbba9frFj0=OqFfea0dXdd9vqai=hGuQ8kuc9pgc9s8qqaq=dirpe0xb9q8qiLsFr0=vr0=vr0dc8meaabaqaciaacaGaaeqabaqabeGadaaakeaacqqHuoardaqhaaWcbaGaemODayNaeGymaeJaeiOla4IaeGOnayJaeGynauJaeiilaWIaem4zaCMaemyzauMaemOBa4MaemyzauMaemOCaiNaemyyaeMaemiBaWgabaGaemODayNaeGymaeJaeiOla4IaeGOnayJaeG4naCJaeiilaWIaem4AaSMaemOBa4Maem4Ba8Maem4DaCNaemOBa4gaaaaa@4AFB@	4735	Δv1.65,non−redundantv1.67,known MathType@MTEF@5@5@+=feaafiart1ev1aaatCvAUfKttLearuWrP9MDH5MBPbIqV92AaeXatLxBI9gBaebbnrfifHhDYfgasaacH8akY=wiFfYdH8Gipec8Eeeu0xXdbba9frFj0=OqFfea0dXdd9vqai=hGuQ8kuc9pgc9s8qqaq=dirpe0xb9q8qiLsFr0=vr0=vr0dc8meaabaqaciaacaGaaeqabaqabeGadaaakeaacqqHuoardaqhaaWcbaGaemODayNaeGymaeJaeiOla4IaeGOnayJaeGynauJaeiilaWIaemOBa4Maem4Ba8MaemOBa4MaeyOeI0IaemOCaiNaemyzauMaemizaqMaemyDauNaemOBa4MaemizaqMaemyyaeMaemOBa4MaemiDaqhabaGaemODayNaeGymaeJaeiOla4IaeGOnayJaeG4naCJaeiilaWIaem4AaSMaemOBa4Maem4Ba8Maem4DaCNaemOBa4gaaaaa@52F9@	302
Δv1.67,generalv1.69,known MathType@MTEF@5@5@+=feaafiart1ev1aaatCvAUfKttLearuWrP9MDH5MBPbIqV92AaeXatLxBI9gBaebbnrfifHhDYfgasaacH8akY=wiFfYdH8Gipec8Eeeu0xXdbba9frFj0=OqFfea0dXdd9vqai=hGuQ8kuc9pgc9s8qqaq=dirpe0xb9q8qiLsFr0=vr0=vr0dc8meaabaqaciaacaGaaeqabaqabeGadaaakeaacqqHuoardaqhaaWcbaGaemODayNaeGymaeJaeiOla4IaeGOnayJaeG4naCJaeiilaWIaem4zaCMaemyzauMaemOBa4MaemyzauMaemOCaiNaemyyaeMaemiBaWgabaGaemODayNaeGymaeJaeiOla4IaeGOnayJaeGyoaKJaeiilaWIaem4AaSMaemOBa4Maem4Ba8Maem4DaCNaemOBa4gaaaaa@4B03@	2298	Δv1.67,non−redundantv1.69,known MathType@MTEF@5@5@+=feaafiart1ev1aaatCvAUfKttLearuWrP9MDH5MBPbIqV92AaeXatLxBI9gBaebbnrfifHhDYfgasaacH8akY=wiFfYdH8Gipec8Eeeu0xXdbba9frFj0=OqFfea0dXdd9vqai=hGuQ8kuc9pgc9s8qqaq=dirpe0xb9q8qiLsFr0=vr0=vr0dc8meaabaqaciaacaGaaeqabaqabeGadaaakeaacqqHuoardaqhaaWcbaGaemODayNaeGymaeJaeiOla4IaeGOnayJaeG4naCJaeiilaWIaemOBa4Maem4Ba8MaemOBa4MaeyOeI0IaemOCaiNaemyzauMaemizaqMaemyDauNaemOBa4MaemizaqMaemyyaeMaemOBa4MaemiDaqhabaGaemODayNaeGymaeJaeiOla4IaeGOnayJaeGyoaKJaeiilaWIaem4AaSMaemOBa4Maem4Ba8Maem4DaCNaemOBa4gaaaaa@5301@	263

**Table 4:** The sequence redundancy in a set that contains 10 pairs of proteins, which are randomly sampled from Δv1.67,non−redundantv1.69,known
 MathType@MTEF@5@5@+=feaafiart1ev1aaatCvAUfKttLearuWrP9MDH5MBPbIqV92AaeXatLxBI9gBaebbnrfifHhDYfgasaacH8akY=wiFfYdH8Gipec8Eeeu0xXdbba9frFj0=OqFfea0dXdd9vqai=hGuQ8kuc9pgc9s8qqaq=dirpe0xb9q8qiLsFr0=vr0=vr0dc8meaabaqaciaacaGaaeqabaqabeGadaaakeaacqqHuoardaqhaaWcbaGaemODayNaeGymaeJaeiOla4IaeGOnayJaeG4naCJaeiilaWIaemOBa4Maem4Ba8MaemOBa4MaeyOeI0IaemOCaiNaemyzauMaemizaqMaemyDauNaemOBa4MaemizaqMaemyyaeMaemOBa4MaemiDaqhabaGaemODayNaeGymaeJaeiOla4IaeGOnayJaeGyoaKJaeiilaWIaem4AaSMaemOBa4Maem4Ba8Maem4DaCNaemOBa4gaaaaa@5301@

*pairs*	*pdb*_*id*_1_	*super family*_*id*_1_	*pdb*_*id*_2_	*super family*_*id*_2_	sequence identity	sequence similarity
01	1*osd*_*A*	55008	1*uta*_*A*	110997	2.10%	3.50%
02	1*ug*8_*A*	82708	1*vm*0_*A*	82704	12.80%	26.80%
03	1*v*5*n*_*A*	57889	1*rq*8_*A*	75471	13.60%	23.50%
04	1*veu*_*B*	103196	1*j*3*m*_*A*	103247	22.40%	34.20%
05	1*tu*1_*B*	55724	1*smb*_*A*	55797	6.80%	10.80%
06	1*thq*_*A*	56925	1*xfs*_*B*	55961	18.10%	28.40%
07	1*vki*_*B*	55826	1*sk*3_*A*	55846	17.70%	30.50%
08	1*tf*1_*D*	55781	1*pp*6_*E*	55676	10.30%	17.50%
09	1*ucd*_*A*	55895	1*vkw*_*A*	55469	9.00%	14.70%
10	1*tt*4_*A*	55931	1*vkp*_*A*	55909	12.70%	21.80%
					Avg. 12.55%	Avg. 21.17%

**Table 5:** Local features of proteins from the SCOP folds: (1)*Heme-dependent peroxidases *: 1*stq*_*A*, 1*sog*_*A*, (2) *Acid proteases*: 1*lee*_*A*, 1*lf*2_*A*. Histogram [a,b] denotes the distance histogram for the *a*^*th *^band region and the *b*^*th *^grayscale bin.

Image Features	1stq_A	1sog_A	1lee_A	1lf2_A
Histogram [1,1]	0.0000	0.0000	0.0000	0.0000
Histogram [l,2]	0.0002	0.0002	0.0000	0.0000
Histogram [l,3]	0.0018	0.0020	0.0001	0.0002
Histogram [1,4]	0.0050	0.0053	0.0009	0.0011
Histogram [2,1]	0.0000	0.0000	0.0000	0.0000
Histogram [2,2]	0.0000	0.0000	0.0000	0.0000
Histogram [2,3]	0.0023	0.0022	0.0000	0.0001
Histogram [2,4]	0.0044	0.0043	0.0012	0.0010
Histogram [3,1]	0.0000	0.0000	0.0000	0.0000
Histogram [3,2]	0.0004	0.0004	0.0003	0.0004
Histogram [3,3]	0.0020	0.0019	0.0017	0.0019
Histogram [3,4]	0.0092	0.0080	0.0048	0.0055
Histogram [4,1]	0.0000	0.0000	0.0000	0.0000
Histogram [4,2]	0.0006	0.0006	0.0015	0.0012
Histogram [4,3]	0.0040	0.0042	0.0056	0.0053
Histogram [4,4]	0.0132	0.0130	0.0172	0.0166
Histogram [5,1]	0.0000	0.0000	0.0000	0.0000
Histogram [5,2]	0.0014	0.0015	0.0036	0.0035
Histogram [5,3]	0.0124	0.0128	0.0133	0.0134
Histogram [5,4]	0.0423	0.0425	0.0298	0.0304
Histogram [6,1]	0.0203	0.0201	0.0179	0.0180
Histogram [6,2]	0.0392	0.0386	0.0291	0.0289
Histogram [6,3]	0.0503	0.0496	0.0474	0.0485
Histogram [6,4]	0.0845	0.0833	0.0796	0.0795

**Table 6:** Global features of proteins from the SCOP folds: (1)*Heme-dependent peroxidases*: 1*stq*_*A*, 1*sog*_*A*, (2)*Acid proteases*: 1*lee*_*A*, 1*lf*2_*A*.

Image Features	1stq_A	1sog_A	1lee_A	1lf2_A
Dimension	291.00	294.00	331.00	329.00
Binary_Threshold	23.0000	23.0000	26.0000	26.0000
Texture_Energy	0.0155	0.0153	0.0107	0.0107
Texture_Entropy	51.7143	51.8067	54.4426	54.4139
Texture_Homogenity1	2.5344	2.5261	2.2184	2.2192
Texture_Homogenity2	1.7608	1.7529	1.4467	1.4485
Texture_Contrast	0.0027	0.0027	0.0041	0.0041
Texture_Correlation	6.8883	6.8914	6.7682	6.7659
Texture_Cluster_Tendency	0.0387	0.0392	0.0517	0.0515

**Table 7:** A comparison of the three features for proteins in the *novel folds *and the *known folds*.

	(*f*_1_) Esumt MathType@MTEF@5@5@+=feaafiart1ev1aaatCvAUfKttLearuWrP9MDH5MBPbIqV92AaeXatLxBI9gBaebbnrfifHhDYfgasaacH8akY=wiFfYdH8Gipec8Eeeu0xXdbba9frFj0=OqFfea0dXdd9vqai=hGuQ8kuc9pgc9s8qqaq=dirpe0xb9q8qiLsFr0=vr0=vr0dc8meaabaqaciaacaGaaeqabaqabeGadaaakeaacqWGfbqrdaqhaaWcbaGaem4CamNaemyDauNaemyBa0gabaGaemiDaqhaaaaa@33A2@ (ςF*)	(*f*_2_)*Dist*	(*f*_3_)*S*
*novel folds*	High	High	High
*known folds*	Low	Low	Low

**Table:** Appendix 1 E-Predict Algorithm

**Require**: *t*, *R*, *b*, *n*_t_, *λ*
1: ∏ = ∅
2: **for **each protein *p *∈ *R ***do**
3: **if ***p*·*fold *∉ ∏ **then**
4: ∏ = ∏ ∪ {*p*·*fold*}
5: *Count*[*p*·*fold*] ← 1
6: **else**
7: *Count*[*p*·*fold*] ← *Count*[*p*·*fold*] + 1
8: **end if**
9: **end for**
10: **for ***i *← 0 to |∏| - 1 **do**
11: **if ***Count*[*i*] <*n*_*t *_**then**
12: ∏ ← ∏ - {*i*}
13: **end if **
14: Esumt MathType@MTEF@5@5@+=feaafiart1ev1aaatCvAUfKttLearuWrP9MDH5MBPbIqV92AaeXatLxBI9gBaebbnrfifHhDYfgasaacH8akY=wiFfYdH8Gipec8Eeeu0xXdbba9frFj0=OqFfea0dXdd9vqai=hGuQ8kuc9pgc9s8qqaq=dirpe0xb9q8qiLsFr0=vr0=vr0dc8meaabaqaciaacaGaaeqabaqabeGadaaakeaacqWGfbqrdaqhaaWcbaGaem4CamNaemyDauNaemyBa0gabaGaemiDaqhaaaaa@33A2@[*i*] ← 0
15: *Count*[*i*] ← 0
16: **end for**
17: **for **each candidate SCOP fold *F*∈ ∏ **do**
18: **for **each *p *∈ *R *starting from the top ranked protein **do**
19: **if ***p*·*fold *= *F ***then**
20: *Count*[*F*] ← *Count*[*F*] + 1
21: **if ***Count*[*F*] <*n*_*t *_**then**
22: Esumt MathType@MTEF@5@5@+=feaafiart1ev1aaatCvAUfKttLearuWrP9MDH5MBPbIqV92AaeXatLxBI9gBaebbnrfifHhDYfgasaacH8akY=wiFfYdH8Gipec8Eeeu0xXdbba9frFj0=OqFfea0dXdd9vqai=hGuQ8kuc9pgc9s8qqaq=dirpe0xb9q8qiLsFr0=vr0=vr0dc8meaabaqaciaacaGaaeqabaqabeGadaaakeaacqWGfbqrdaqhaaWcbaGaem4CamNaemyDauNaemyBa0gabaGaemiDaqhaaaaa@33A2@ [*F*] ← Esumt MathType@MTEF@5@5@+=feaafiart1ev1aaatCvAUfKttLearuWrP9MDH5MBPbIqV92AaeXatLxBI9gBaebbnrfifHhDYfgasaacH8akY=wiFfYdH8Gipec8Eeeu0xXdbba9frFj0=OqFfea0dXdd9vqai=hGuQ8kuc9pgc9s8qqaq=dirpe0xb9q8qiLsFr0=vr0=vr0dc8meaabaqaciaacaGaaeqabaqabeGadaaakeaacqWGfbqrdaqhaaWcbaGaem4CamNaemyDauNaemyBa0gabaGaemiDaqhaaaaa@33A2@ [*F*] + *E*_*Measure*(*p*, *b*)
23: **end if **
24: **end if **
25: **end for**
26: **end for**
27: F* ← arg min_*f *_Esumt MathType@MTEF@5@5@+=feaafiart1ev1aaatCvAUfKttLearuWrP9MDH5MBPbIqV92AaeXatLxBI9gBaebbnrfifHhDYfgasaacH8akY=wiFfYdH8Gipec8Eeeu0xXdbba9frFj0=OqFfea0dXdd9vqai=hGuQ8kuc9pgc9s8qqaq=dirpe0xb9q8qiLsFr0=vr0=vr0dc8meaabaqaciaacaGaaeqabaqabeGadaaakeaacqWGfbqrdaqhaaWcbaGaem4CamNaemyDauNaemyBa0gabaGaemiDaqhaaaaa@33A2@[*f*]
28: **if **(λ = *on*) AND (S(*t*,*P*_0_) < S(*t*,PNNF* MathType@MTEF@5@5@+=feaafiart1ev1aaatCvAUfKttLearuWrP9MDH5MBPbIqV92AaeXatLxBI9gBaebbnrfifHhDYfgasaacH8akY=wiFfYdH8Gipec8Eeeu0xXdbba9frFj0=OqFfea0dXdd9vqai=hGuQ8kuc9pgc9s8qqaq=dirpe0xb9q8qiLsFr0=vr0=vr0dc8meaabaqaciaacaGaaeqabaqabeGadaaakeaacqWGqbaudaqhaaWcbaGaemOta4KaemOta4eabaGaemOrayKaeiOkaOcaaaaa@323D@)) **then**
29: F* ← *P*_0.*fold*_
30: **end if **
31: **return **F*
